# BEL1-like Homeodomain Protein BLH6a Is a Negative Regulator of *CAld5H2* in Sinapyl Alcohol Monolignol Biosynthesis in Poplar

**DOI:** 10.3389/fpls.2021.695223

**Published:** 2021-06-25

**Authors:** Qiao Wang, Xinren Dai, Hongying Pang, Yanxia Cheng, Xiong Huang, Hui Li, Xiaojing Yan, Fachuang Lu, Hairong Wei, Ronald R. Sederoff, Quanzi Li

**Affiliations:** ^1^State Key Laboratory of Tree Genetics and Breeding, Chinese Academy of Forestry, Beijing, China; ^2^Research Institute of Forestry, Chinese Academy of Forestry, Beijing, China; ^3^Department of Energy Great Lakes Bioenergy Research Center, Wisconsin Energy Institute, Madison, WI, United States; ^4^College of Forest Resources and Environmental Science, Michigan Technological University, Houghton, MI, United States; ^5^Forest Biotechnology Group, Department of Forestry and Environmental Resources, North Carolina State University, Raleigh, NC, United States

**Keywords:** lignin, *CAld5H2*, BEL1-like homeodomain protein, transcription factor, yeast one hybrid

## Abstract

Lignin is one of the major components of xylem cell walls in tree stems. The lignin in the wood of most flowering plants (dicotyledonous angiosperms) is typically polymerized from three monolignol precursors, coniferyl alcohol, sinapyl alcohol, and *p*-coumaroyl alcohol, resulting in guaiacyl (G), syringyl (S), and hydroxyphenyl (H) subunits, respectively. In this study, we focus on the transcriptional regulation of a coniferaldehyde 5-hydroxylase (*CAld5H2*) gene, which encodes a key enzyme for sinapyl alcohol biosynthesis. We carried out a yeast one-hybrid (Y1H) screen to identify candidate upstream transcription factors (TFs) regulating *CAld5H2*. We obtained 12 upstream TFs as potential regulators of *CAld5H2*. One of these TF genes, BLH6a, encodes a BEL1-like homeodomain (BLH) protein and negatively regulated the *CAld5H2* promoter activity. The direct regulation of *CAld5H2* promoter by BLH6a was supported by chromatin immunoprecipitation–quantitative polymerase chain reaction (ChIP–qPCR) and dominant repression of BLH6a in transgenic plants. Luciferase complementation imaging analyses showed extensive protein**–**protein interactions among these 12 TFs. We propose that BLH6a is a negative regulator of *CAld5H2*, which acts through combinatorial regulation of multiple TFs for sinapyl alcohol (S monolignol) biosynthesis in poplar.

## Introduction

Lignin is a polyphenolic polymer deposited in the secondary cell walls (SCWs) of vascular plants (Sarkanen and Ludwig, 1971). Many different phenylpropanoid subunits may be incorporated into lignin, but the wood of trees within the angiosperms typically contains three predominant lignin subunits, hydroxyphenyl (H), guaiacyl (G), and syringyl (S), derived from *p*-coumaryl, coniferyl, and sinapyl alcohols, also called monolignols. Lignin composition depends on the relative abundance of monolignols, which are polymerized by radical coupling reactions (Ralph et al., 2004). In angiosperm wood, typically in dicots, lignin is composed primarily of G and S subunits, with minor amounts of H subunits. The proportion of G and S subunits in lignin is different between cell types. Vessel elements are specialized for water transport, and fiber cells provide mechanical support. In the wood of poplar, guaiacyl lignin is predominant in vessel cell walls, whereas S subunits are mainly located in fiber cell walls (Zhou et al., 2011). In gymnosperms, the mature xylem contains one major type of cell (tracheids), which carries out both support and transport. Tracheid lignin has mainly G subunits with only minor amounts of H subunits in their cell walls (Sarkanen and Ludwig, 1971).

**Figure 1:**
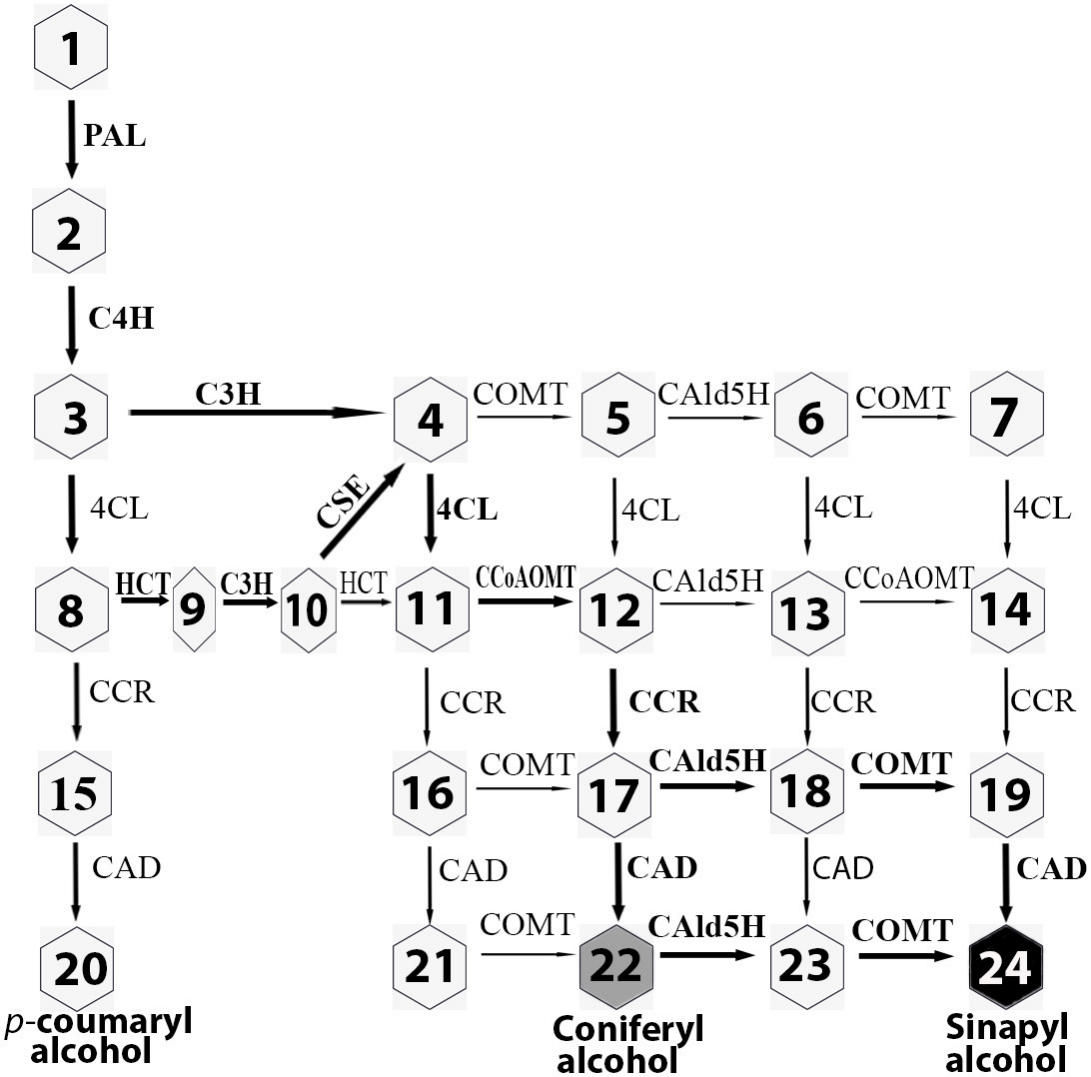
Monolignol biosynthetic pathway in poplar. Bold arrows indicate the main metabolic flux. Eleven enzyme families are PAL, phenylalanine ammonia-lyase; C4H, cinnamate 4-hydroxylase; C3H, *p*-coumaroyl-CoA 3-hydroxylase; 4CL, *p*-coumarate CoA ligase; HCT, hydroxycinnamoyltransferase; CSE, caffeoyl shikimate esterase; CCoAOMT, caffeoyl-CoA *O*-methyltransferase; CCR, cinnamoyl-CoA reductase; CAld5H, coniferaldehyde 5-hydroxylase; COMT, caffeic acid 3-*O*-methyltransferase; CAD, cinnamyl alcohol dehydrogenase. The metabolites are shown in numbers. 1: phenylalanine; 2: cinnamic acid; 3: 4-hydroxycinnamic acid; 4: caffeic acid; 5: ferulic acid; 6: 5-hydroxyferulic acid; 7: sinapic acid; 8: *p*-coumaroyl-CoA; 9: *p*-coumaroyl shikimic acid; 10: caffeoyl shikimic acid; 11: caffeoyl-CoA; 12: feruloyl-CoA; 13: 5-dydroxyferuloyl-CoA; 14: sinapoyl-CoA; 15: *p*-coumaraldehyde; 16: caffeyl aldehyde; 17: coniferaldehyde; 18: 5-hydroxyconiferaldehyde; 19: sinapaldehyde; 20: *p*-coumaryl alcohol (H monolignol); 21: caffeyl alcohol; 22: coniferyl alcohol (G monolignol); 23:5-hydroxyconiferyl alcohol; 24: sinapyl alcohol (S monolignol).

In angiosperms, 11 enzyme families comprise a branched grid-like pathway that converts phenylalanine to three major monolignols (Boerjan et al., 2003; Vanholme et al., 2013) (Figure 1). These enzyme families are designated PAL (phenylalanine ammonia-lyase, EC 4.3.1.5), C4H (cinnamate 4-hydroxylase, EC 1.14.13.11), C3H (*p*-coumaroyl-CoA 3-hydroxylase, EC 1.14.14.1), 4CL (*p*-coumarate CoA ligase, EC 6.2.1.12), HCT (hydroxycinnamoyltransferase, EC 2.3.1.133), CSE (caffeoyl shikimate esterase, EC 3.1.1-), CCoAOMT (caffeoyl-CoA *O*-methyltransferase, EC 2.1.1.104), CCR (cinnamoyl-CoA reductase, EC 1.2.1.44), F5H/CAld5H [ferulate/coniferaldehyde 5-hydroxylase (CAld5H2), EC 1.14.13], COMT (caffeic acid 3-*O*-methyltransferase, EC 2.1.1.6), and CAD (cinnamyl alcohol dehydrogenase, EC 1.1.1.95). In this pathway, coniferaldehyde is converted to coniferyl alcohol by CAD for G monolignol production. Alternatively, coniferaldehyde can be converted to sinapyl alcohol in three steps by three consecutive enzymes, CAld5H/F5H, COMT, and CAD. CAld5H/F5H is a key enzyme in directing S lignin production (Osakabe et al., 1999). Overexpression of *F5H* driven by a *C4H* promoter caused an increase (29.5%) in sinapyl alcohol level in hybrid poplar (Stewart et al., 2009).

Many transcription factors (TFs) in the transcriptional network regulating the SCW synthesis have been characterized (Ko et al., 2014). In the top layer of the gene regulatory network (GRN), NAC [no apical meristem (NAM), arabidopsis transcription activator factor (ATAF1/2), and cup-shaped cotyledon (CUC)] and v-myb avian myeloblastosis viral oncogene homolog (MYB) members, such as secondary wall-associated NAC domain protein1/vascular-related NAC-domain6 (SND1/VND6) (Minoru et al., 2005; Zhong et al., 2006) and MYB46/83 (Zhong et al., 2007; McCarthy et al., 2009; Zhong and Ye, 2012), are master regulators activating SCW synthesis. Most TFs in the GRN are activators and a few are repressors, indicating fine-tuning of the transcriptional control in SCW biosynthesis (Ko et al., 2014). Some TFs in the network directly regulate genes encoding pathway enzymes for cellulose, hemicellulose, and lignin biosynthesis (Ohashiito et al., 2010; Kim et al., 2013). The TFs that directly regulate lignin biosynthesis could act to differentially activate or repress lignin pathway genes. For example, MYB46 directly regulates nine of the 10 *Arabidopsis* lignin pathway genes characterized (Ko et al., 2014), whereas MYB58 directly activates all 10 of the *Arabidopsis* lignin pathway genes (Zhou et al., 2009). In Medicago, SND1 directly activates *F5H*, but the SND1 in *Arabidopsis* does not (Zhong et al., 2008; Zhao et al., 2010), indicating that the GRN for SCW synthesis may not be the same in different plant species.

Wood provides large quantities of material for the production of pulp, paper, timber, and lignocellulosic biofuels. For pulp, paper, and chemical feedstock production, lignin is a major barrier to such applications and must be removed. The content, composition, and structure of lignin affect the process efficiency of woody biomass (Studer et al., 2011; Li et al., 2014). Lignin composition, calculated as the S/G ratio, is an important factor affecting the pulp yield than the lignin content (Río et al., 2005; Studer et al., 2011). High pulp yield is correlated with a high S/G ratio (Río et al., 2005). Hardwood is generally delignified more readily than softwood due to the abundance of S subunits in the hardwood lignin (Sarkanen and Ludwig, 1971; Chang and Sarkanen, 1973). Wood with a higher proportion of S subunits is preferred for paper/pulping and biofuel production (Wagner et al., 2015).

The favorable properties of lignin with a higher S subunit content have motivated efforts to increase the S lignin content in wood through genetic modification. Overexpression of *CAld5H* had a 64% increase in the S/G ratio in poplar (Li et al., 2003). Lignin polymers that contain S units have been generated in conifer cells by overexpression of *F5H* in *Pinus radiata* tracheary element cultures. Co-transformation of *F5H* and *COMT* resulted in a two to three times higher S/G ratio than *F5H* alone (Wagner et al., 2015).

To learn more about the GRN that determines SCW biosynthesis, we identified and characterized TFs regulating sinapyl alcohol (monolignol) biosynthesis. We conducted a yeast one hybridization (Y1H) screening of a library of TFs from *Populus trichocarpa*, using the *CAld5H2* promoter as bait, and identified 12 candidates as potential targets. Among the 12 TFs, two BEL1-like homeodomain (BLH) 6 proteins, BLH6a and BLH6b, bound *CAld5H2* promoter specifically.

We characterized the functions of BLH6a. Transient overexpression and dominant repression of *BLH6a* regulation inhibited *CAld5H2* expression, and extensive protein**–**protein interactions were detected among the 12 TFs. Our results suggest that a complex GRN with combinatorial and redundant elements may control the *CAld5H* gene expression for S lignin biosynthesis.

## Materials and Methods

### Plant Materials

*Populus alba* × *Populus glandulosa* and *Nicotiana benthamiana* plants were grown in a room with long-day conditions (16-h light/8-h dark) at 25 ± 1°C. The *P. alba* × *P. glandulosa* sterile plants used for transformation were propagated by microcuttings in bottles and cultured on 0.5 × the Murashige and Skoog (MS) medium.

### Yeast One-Hybrid

Total RNAs were extracted from differentiating xylem of 6-month-old *P. trichocarpa* trees using an RNeasy Plant Mini Kit (QIAGEN Inc., Valencia, CA, USA) and reverse-transcribed to complementary DNAs (cDNAs) by using the PrimeScript™ RT Reagent Kit (TaKaRa, Dalian, China). The cDNAs were used as templates to amplify the coding region of 213 TFs (Supplementary Data Sheet 1) with the Phanta Max Super-Fidelity DNA Polymerase (Vazyme Biotech Co., Ltd., Nanjing, Jiangsu, China). About 202 PCR fragments were digested with corresponding restriction enzymes and cloned into the pGADT7 vectors by T4 DNA ligases. About 11 PCR fragments were cloned into the pGADT7 vector through the recombination method by using the ClonExpressII One Step Cloning Kit (Vazyme Biotech Co., Ltd., Nanjing, Jiangsu, China). The full lengths of coding regions cloned in pGADT7 were sequence confirmed. The coding regions of 14 TFs were synthesized and cloned into pGADT7. Total 227 TF-prey plasmids were constructed.

Genomic DNA was extracted from young leaves using a DNeasy Plant Mini Kit (QIAGEN Inc., Valencia, CA, USA). Gene promoter regions were amplified from genomic DNA and cloned into the pABAi vector by T4 DNA ligases. The resultant DNA-bait plasmid was linearized by *Bstb*I and integrated into the Y1HGold Strain (Clontech Laboratories, Inc., Mountain View, CA, USA). Four transformants were tested on the SD/-ura medium with the addition of Aureobasidin A (AbA) ranging from 100 to 500 μg/ml, and the DNA-bait strain with optimal AbA concentration was selected for Y1H.

The 227 TF-prey plasmids were individually transformed into the DNA-bait strain, and the transformants were selected on the SD/-ura-leu medium. The positive strains were diluted with 0.9% NaCl and screened on the SD/-ura-leu+AbA medium for identifying the TF–DNA interaction.

### Effector-Reporter-Based Transactivation/Repression Assays

The 2-kb *CAld5H2* promoter was amplified from *P. alba* × *P. glandulosa* genomic DNA and cloned into *pBG3-LZ004-LUC* (luciferase) (Chen et al., 2017) by using the ClonExpressII One Step Cloning Kit (Vazyme Biotech Co., Ltd., Nanjing, Jiangsu, China), generating the reporter construct *pBG3-pCAld5H2:LUC*. The full-length coding regions of *BLH6a, BLH6b*, and *BLH2* were amplified from *P. alba* × *P. glandulosa* xylem cDNA and cloned into the pENTR/D TOPO Vector (Invitrogen Co., Carlsbad, CA, USA). After being verified by sequencing, the genes were Gateway cloned into the pGWB17 Vector by LR Clonase II (Invitrogen), generating the effector constructs *pBWB17-35S:BLH6a, pBWB17-35S:BLH6b*, and *pBWB17-35S:BLH2*. The resultant reporter constructs and effector constructs were transformed into *Agrobacterium* GV3101. *Agrobacterium* containing an effector construct and a reporter construct were co-injected into the tobacco leaves. After 48 h, the LUC luminescence was detected under a Promega GloMax^®^-20/20 Luminometer (Promega Corp., Madison, WI, USA). The primers used for the promoter and gene amplification are listed in Supplementary Table 1.

### Transcriptional Activation/Inhibition Assays in Yeast

The full-length coding region of *BLH6a* was amplified from *P. alba* × *P. glandulosa* xylem cDNA with the Phanta Max Super-Fidelity DNA Polymerase (Vazyme Biotech Co., Ltd., Nanjing, Jiangsu, China) and cloned into pGBKT7 and pGBKT7-VP16 vector using the ClonExpressII One Step Cloning Kit (Vazyme Biotech Co., Ltd., Nanjing, Jiangsu, China), generating BD-BLH6 and BD-VP16:BLH6a. The constructs were transformed into the yeast strain, yeast two hybridization, Y2H Gold (Clontech Laboratories, Inc., Mountain View, CA, USA). Transformants were grown on the SD/-Trp medium for the selection of positive clones and then transferred to the SD/-Trp-His-Ade medium for the transcriptional activation/inhibition assays with X-α-gal used as an indicator.

### Chromatin Immunoprecipitation–Quantitative PCR Assay

The fragment of 3× FLAG was amplified from the *pCM1307-N-Flag-HA vector* (Zhou et al., 2012), cloned into the pENTR/D TOPO Vector (Invitrogen), and LR ligated into *pUC19-35S-RfA-35S-sGFP* (Li et al., 2012) to generate *pUC19-35S-FLAG-35S-sGFP*. BLH6a coding sequences were amplified from *P. alba* × *P. glandulosa* xylem cDNA and cloned into *pUC19-35S-FLAG-35S-sGFP*, generating *pUC19-35S-Flag:BLH6a-35S-sGFP*. Primers are listed in Supplementary Table 1. Plasmid DNA for the protoplast transfection was prepared using the EndoFree Plasmid Kit (QIAGEN Inc., Valencia, CA, USA). Leaf protoplasts were prepared from mesophyII of leaves in tissue culture bottles following the published protocol (Lee et al., 2017), and xylem protoplasts were prepared as described previously (Lin et al., 2014). Plasmids were transferred into protoplasts, and each chromatin immunoprecipitation (ChIP) assay was performed using 5 × 10^6^ protoplasts with anti-FLAG antibodies as described before (Yan et al., 2019). One-fiftieth of the supernatants before adding antibodies were used as input in the qPCR. qPCR was conducted using the Green Premix Ex Tag II (TaKaRa, Dalian, China) and detected by the Roche LightCycler 480 II (Roche Co., Basel, Switzerland), with 18S as the reference.

### RNA *in-situ* Hybridization

The eighth and nineth internodes of 3-month-old *P. alba* × *P. glandulosa* stems were fixed in formalin/acetic acid/alcohol (FAA) and embedded in paraplast. The 8 μm microsections were prepared for the *in situ* hybridization. PCR fragments of ~250 bp, amplified with gene-specific primers (Supplementary Table 1), were used as templates to synthesize antisense and sense probes with T7 RNA polymerases using the Digoxigenin (DIG) RNA Labeling Kit (Roche). The hybridization and immunological detection were performed as previously described (Liu et al., 2018). The hybridization solution contains 10× *in situ* salts, deionized formamide, 50% dextran sulfate, 50× Denhardt's solution, and 100 ng/ml tRNA. Signals were detected using anti-DIG antibodies conjugated with alkaline phosphatase, and photographs were taken under an OLYMPUS BX51 Microscope (Olympus Corp., Tokyo, Japan).

### Yeast Two Hybridization

The full-length coding regions of *BLH6b* and *BLH2* were amplified from *P. alba* × *P. glandulosa* xylem cDNA with the Phanta Max Super-Fidelity DNA Polymerase (Vazyme Biotech Co., Ltd., Nanjing, Jiangsu, China) and cloned into the pGADT7 vector by using the ClonExpressII One Step Cloning Kit (Vazyme Biotech Co., Ltd., Nanjing, Jiangsu, China), generating AD fusion vectors. The full-length coding region of *BLH6a* was cloned into the pGBKT7 vector, generating the BD-BLH6a vector. The BD-BLH6a and AD fusion vectors were co-transformed into the yeast strain *Saccharomyces cerevisiae* Y2HGold as described in the Yeast Protocols Handbook (Clontech Laboratories, Inc., Mountain View, CA, USA). Transformants were grown on the SD/-Leu-Trp medium and then screened on the SD/-Ade-His-Leu-Trp medium.

### Luciferase Complementation Imaging Assays

The coding region of 12 TFs was amplified from *P. alba* × *P. glandulosa* xylem cDNA, and, by using the ClonExpressII One Step Cloning Kit (Vazyme Biotech Co., Ltd., Nanjing, Jiangsu, China), they were cloned into *pCAMBIA1300-cLUC* and *pCAMBIA1300-nLUC*, which were pre-linearized by *Kpn*I/*Sal*I (Song et al., 2011), generating *TF-nLUC* and *TF-cLUC*. Primers used for the vector construction are shown in Supplementary Table 1. After confirmation by sequencing, the vectors were transformed into *Agrobacteriu*m GV3101. Equal concentrations and volumes of *Agrobacterium* cultures were mixed and co-infiltrated into the *N. benthamiana* leaves. After incubation for 36 h, the LUC fluorescence was detected under a Berthold NightSHADE LB985 *in vivo* Plant Imaging System (Berthold Technologies GmbH, Bad Wildbad, Germany).

### Vector Construction and Transgenic Production

Two oligos EAR-motif repression domain (SRDX)-F and SRDX-R (Supplementary Table 1) were synthesized. After denature and annealing, the double-strand DNA fragment was ligated into *pBI121* at *BamH*I/*Sac*I, generating *pBI121-35S-SRDX*. This vector contains a short sequence between 35S promoter and stops codon, encoding a 12 amino acid (LDLDLELRLGFA) plant-specific SRDX (Mitsuda et al., 2011).

The full-length coding region of *BLH6a* was amplified by 121BLH6a-F/R from *P. alba* × *P. glandulosa* xylem cDNA and inserted into *pBI121-35S-SRDX* using the ClonExpressII One Step Cloning Kit (Vazyme Biotech Co., Ltd., Nanjing, Jiangsu, China), generating *pBI121-35S-BLH6a:SRDX*. The resultantly plasmid was transformed into *Agrobacterium* GV3101, and transformation in *P. alba* × *P. glandulosa* was conducted by the leaf disk method. The leaves were cut at vein area, immersed in *Agrobacterium* culture for 15 min. After incubated on the co-cultivation medium (1× MS, pH 5.7–5.9, 5 mg/L 6BA, 0.05 mg/L NAA) in dark for 4 days, the explants were transferred to the selection medium (1× MS, pH 5.7–5.9, 0.5 mg/L 6BA, 0.05 mg/L NAA, 200 mg/L timentin, 50 mg/L kanamycin) under light with the medium substitution every 2 weeks. The generated shoots were cut and transferred to the rooting medium (0.5 × MS, pH 5.7–5.9, 0.2 mg/L NAA, 0.05 mg/L IBA, 200 mg/L timentin, 50 mg/L kanamycin) for the root induction.

### Quantitative Reverse Transcription-PCR

Total RNAs were extracted from differentiating xylem of 6-month-old trees using the RNeasy Plant Mini Kit (QIAGEN Inc., Valencia, CA, USA). The total RNAs were reverse transcribed to cDNA using a PrimeScript™ RT Reagent Kit with gDNA Eraser (TaKaRa, Dalian, China). The PCR was conducted using the Green Premix Ex Tag II (TaKaRa, Dalian, China) and detected by the Roche LightCycler 480 II. Actin was used as the reference.

### Stem Sectioning and Light Microscopy

The stem fragments, close to the bottom node, were collected from the seventh to 14th internodes, immobilized with glue (LOCTITE 495), and sectioned by an oscillating Leica VT 1000 S Microtome (Leica Microsystems, Wetzlar, Germany). The section thickness was 40 μm. The sections were stained in 0.05% (w/v) toluidine blue (TBO) dye for 1 min and observed under an OLYMPUS BX51 Microscope (Olympus Corp., Tokyo, Japan).

### Lignin Content and Composition

The stems below the 20th internode were collected from 9-month-old trees, and bark was removed. Dried wood was ground, lyophilized, and extracted with chloroform/methanol (2:1, v/v). The lignin content was determined by the Klason method as described before (Lu et al., 2013). Lignin compositions were determined by the thioacidolysis method (Lapierre et al., 1995).

## Results

### Identification of 12 TF Candidates That Bound the *P. trichocarpa cAld5H2* Promoter

In *P. trichocarpa*, two closely related genes *CAld5H1* (Potri.005G117500) and *CAld5H2* (Potri.007G016400) are key genes in S monolignol biosynthesis with redundant functions (Wang et al., 2012). These enzymes comprise a key branch point in monolignol biosynthesis because they regulate the relative abundance of substrate that becomes coniferyl alcohol as opposed to 5-hydroxyconiferyl alcohol. These enzymes act on coniferyl alcohol directly or they may act on the aldehyde intermediates converting coniferylaldehyde to 5-hydroxyconiferaldehyde. The synthesis of 5-hydroxy intermediates determines the amount of sinapylaldehyde and sinapyl alcohol that is formed and therefore the relative abundance of sinapyl and coniferyl alcohol. The ratio of these alcohols is a major determinant of the ratio of S and G subunits polymerized into lignin (Osakabe et al., 1999; Wang et al., 2014, 2018).

We conducted a Y1H screening to identify TFs that could directly regulate *CAld5H* genes. Using our previous RNA-seq data (Shi et al., 2017) from xylem, phloem, leaves, and shoot tips from *P. trichocarpa*, we obtained 227 TFs differentially expressed in xylem compared to three other tissues [fold change >1, and false discovery rate (FDR) <0.05] (Supplementary Data Sheet 1). We generated a TF-prey library by cloning 227 TFs into the yeast expression vector pGADT7. We isolated the 2 kb promoter of *CAld5H2* from *P. trichocarpa* genomic DNA and used it as bait to screen the TF-prey library. The 227 TF-prey plasmids were individually transformed into the bait-strain and selected on the SD/-leu-ura medium containing AbA, we obtained 57 TF containing clones that could grow on the selective medium.

To eliminate the false positives from the 57 TF candidates, we performed co-expression analysis of 57 TFs with *CAld5H* genes using the AspWood web resource (http://aspwood.popgenie.org). AspWood provides a high-spatial-resolution gene expression profile of 25–28 stem cryosections in *P. tremula*, from phloem to cambium and then to wood-forming tissues, enabling the co-expression network analysis (Sundell et al., 2017). Of the 57 TF genes that passed the screen, 12 were co-expressed with both *CAld5H2 and CAld5H1* transcripts (Supplementary Figure 1, Supplementary Table 2). Their expression levels were low in the cambium (sections 5–9). Starting from section 9 in the wood-forming tissue, their expression levels gradually increased and were maintained at a higher level (Supplementary Figure 2), indicating their roles in xylem cell differentiation and SCW biosynthesis during wood formation. We chose these 12 TFs for further characterization.

**Figure 2:**
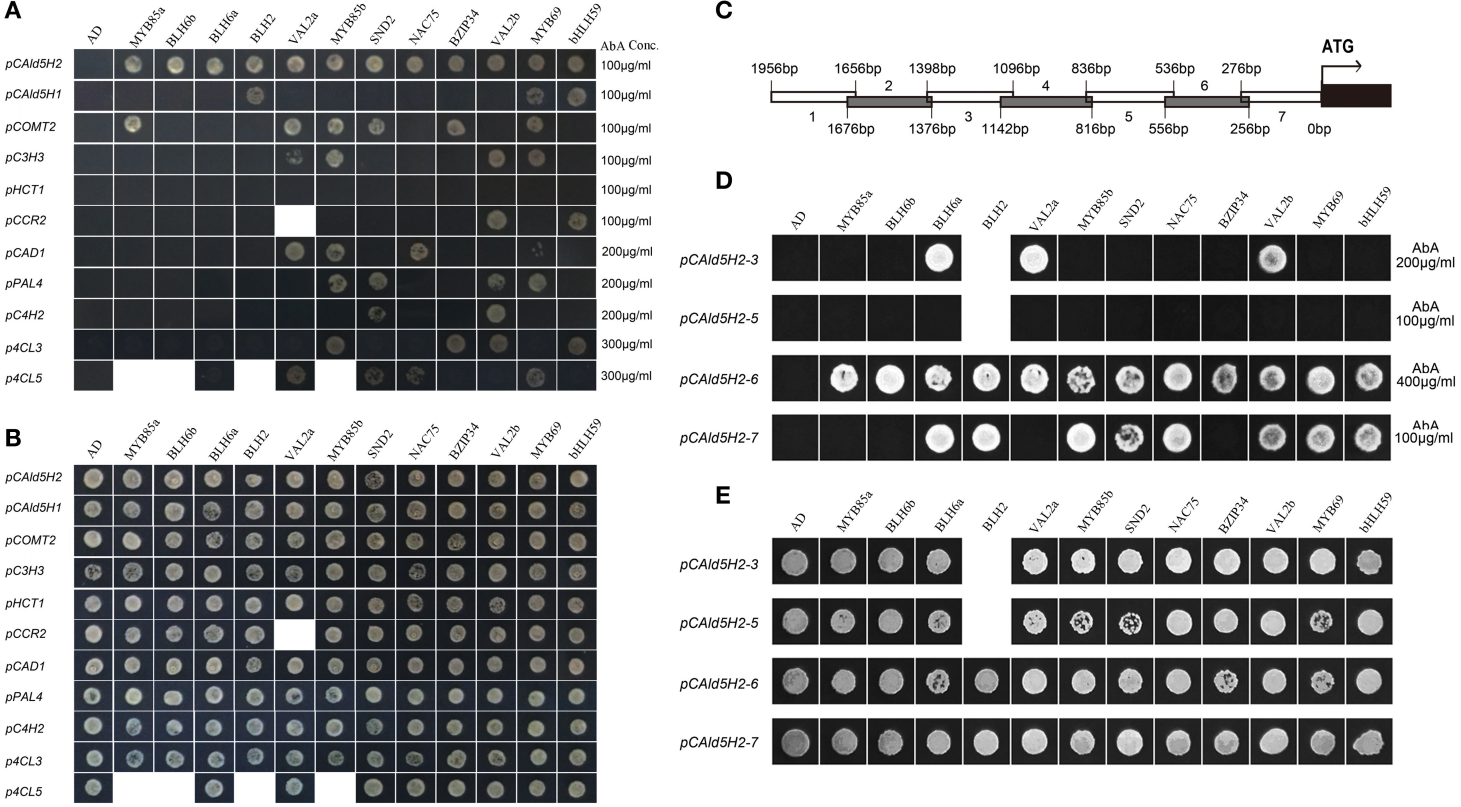
Yeast one hybridization. **(A,B)** Yeast one-hybrid (Y1H) of 12 transcription factors (TFs) with 11 monolignol pathway genes in *Populus trichocarpa*. TF-prey plasmids were transformed into bait strains and grown on SD/-ura-leu. We did not obtain transformants for *VAL2a* transformed into *CCR2* promoter strain, and for *MYB85a, BLH6b, BLH2*, and *MYB85b* transformed into *4CL5* promoter strain **(B)**. The transformants were selected on SD/-ura-leu+AbA **(A)**. The concentrations of Aureobasidin A (AbA) are shown on right. **(C–E)** Y1H of 12 TFs with seven fragments of *CAld5H2* promoter in *P. trichocarpa*. **(C)** The 2 kb *P. trichocarpa CAld5H2* promoter was divided into seven fragments. Each fragment is about 300 bp long and two adjacent fragments have a 20–46 bp overlap. **(D)** Y1H assays of 12 TFs with fragments (3, 5, 6, and 7) of *CAld5H2* promoters. The self-activation of fragments 1, 2, and 4 are too higher (1,000 μg/ml) to be removed. The transformants were grown on SD/-ura-leu+AbA for selection. The AbA concentrations were shown on the right. **(E)** The transformants were grown on SD/-ura-leu.

To identify how many TFs among these 12 could interact with the *CAld5H2* promoter among the monolignol pathway gene promoters, we conducted Y1H assays to compare these 12 TFs with other monolignol pathway gene promoters isolated from *P. trichocarpa*, including *CAD1, C3H3, COMT2, CCR2, PAL4, HCT1, 4CL3, 4CL5*, and *C4H2*. Among these 12 TFs, only the homologs of BLH6a (Potri.004G159300) and BLH6b (Potri.009G120800) bound *CAld5H2* promoter specifically. BLH2 (Potri.005G129500) bound both *CAld5H2* and *CAld5H1* promoters, but not other promoters (Figures 2A,B). This three TFs belong to the BLH family, indicating that the BLH protein family may be more specific in binding to *CAld5H* promoters. The other nine TFs recognized promoters of other genes in the monolignol pathway (Figures 2A,B), suggesting that they regulate other steps in monolignol biosynthesis.

### All 12 Selected TFs Bound the Fragments 6 and 7 of the *CAld5H2* Promoter

To identify the specific binding regions of these 12 TFs in the *CAld5H2* promoter in *P. trichocarpa*, we divided the 2 kb promoter into seven fragments (Figure 2C). The Y1H assays were conducted to examine the interactions of 12 TFs with the seven fragments. The self-activation in the bait strains containing fragments 1, 2, and 4 was very strong, and therefore these fragments were removed in the study. The Y1H assays showed that all 12 TFs could bind fragment 6 of *CAld5H2* promoter, and eight TFs could bind fragment 7. Fragment 3 of the promoter was recognized by BLH6a, VAL2a, and VAL2b. None of the TFs bound to fragment 5 (Figure 2D).

Fragments 6 and 7 are major binding sites for all 12 TFs. We used PlantPAN 3.0 (http://plantpan.itps.ncku.edu.tw/index.html) to characterize the specificity of cis-acting regulatory DNA elements and to identify possible binding to TF families. Besides the CAAT box and TATA box, multiple cis-acting regulatory elements were found, including WBOXATNPR1, MYBCOREATCYCB1, DOFCOREZM, and SURECOREATSULTR11 (Supplementary Data Sheet 2). The possible TF families that could bind to these motifs include AT-HOOK, Dof, ATA, ALE, WOX, MYB, bZIP, bHLH, NAC, HD-ZIP, and ZF-H (Supplementary Data Sheet 2). Fragments 6 and 7 contain motifs for recognition by the families of 12 TFs, such as the “TGAC” element in motif WBOXATNPR1 for BLH proteins, and the “CANNTG” element in YCCONSENSUSAT motif for bHLH and bZIP TFs (Murre et al., 1989; Ledent and Vervoort, 2001; Kondhare et al., 2019). This analysis supports the binding of 12 TFs to the *CAld5H2* promoter.

### *P. alba* × *P. glandulosa* BLH6a Negatively Regulated *CAld5H2* Promoter Activity

Among the three potential *CAld5H2*-specific upstream regulators, BLH2, also named WBLH2 (Chen et al., 2019), has been implicated as an upstream regulator and was shown to bind the *CAld5H1* promoter by ChIP–PCR in *P. trichocarpa* (Chen et al., 2019). Although *BLH6a* (also named WBLH3 (Chen et al., 2019) has been implicated as a target of PtrMYB021, the regulatory function of these two BLH6 members on the monolignol pathway genes was not studied before.

**Figure 3:**
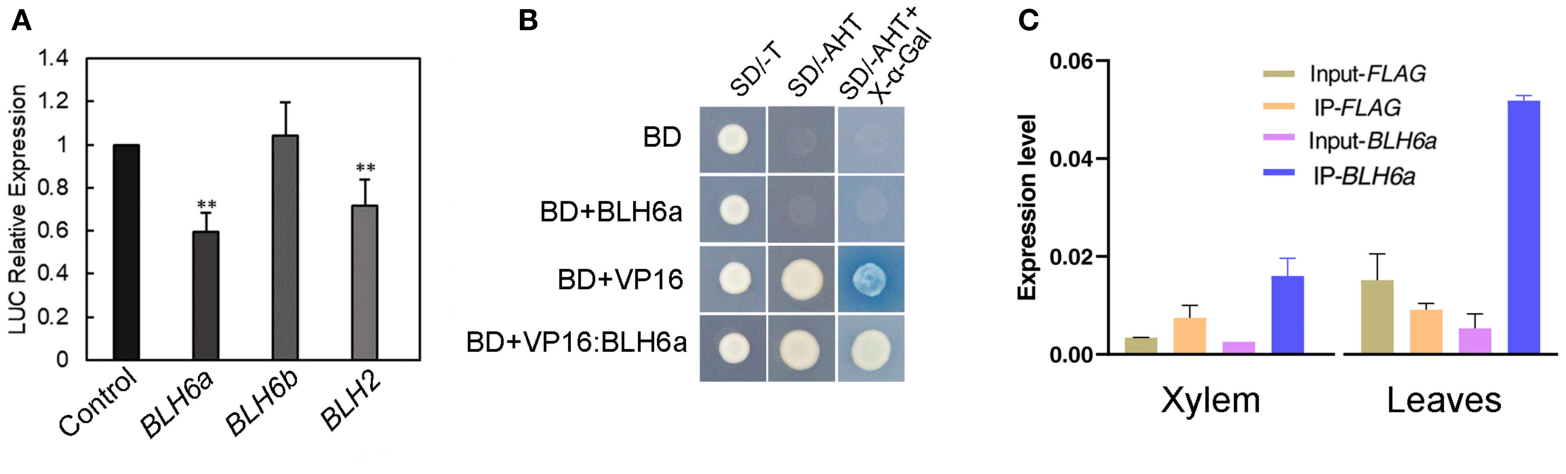
BLH6a is a transcriptional repressor of *CAld5H2* in *P. alba* × *P. glandulosa*. **(A)** Effector-reporter-based gene activation/repression assay. *LUC* gene was driven by the *P. alba* × *P. glandulosa CAld5H2* promoter in the reporter construct. Each TF gene from *P. alba* × *P. glandulosa* was driven by the 35S promoter in the effector construct. Effect construct and reporter construct were co-infiltrated in the tobacco leaves by *Agrobacterium* for the LUC activity determination. The reporter construct alone was the control. **(B)** Transactivation/repressor activity detections in yeast strain Y2HGold. SD/-T, single dropout medium lacking tryptophan; SD/-AHT, triple dropout medium lacking adenine, histidine, and tryptophan; BD, GAL4-binding domain. **(C)** Chromatin immunoprecipitation (ChIP) assays in xylem (left) and leaf (right) protoplasts. ChIP was conducted in protoplasts overexpressing *FLAG* and *FLAG:BLH6a*, respectively, and followed by qPCR. Input-FLAG and Input-BLH6a were IP using one-fiftieth of the supernatants before adding antibodies in the *FLAG* and *FLAG:BLH6a* overexpression protoplasts, respectively. IP-FLAG and IP-BLH6a were IP with FLAG antibodies in *FLAG* and *FLAG:BLH6a* overexpression protoplasts, respectively. Error bars represent SD (*n* = 3). **p < 0.01, determined by Student's t test.

To investigate whether the two BLH6 homologs regulate *CAld5H2 in vivo*, we conducted effector-reporter-based transactivation/repression assays. The reporter constructs, carrying a *LUC* luciferase gene under the control of the *P. alba* × *P. glandulosa CAld5H2* promoter and was co-expressed transiently in *N. benthamiana* leaves with effector construct harboring either *BLH6a* or *BLH6b* from the *P. alba* × *P. glandulosa* hybrid. BLH6a repressed reporter activity, but BLH6b did not (Figure 3A). BLH2 also inhibited *CAld5H2* promoter activity (Figure 3A). We further used the yeast system to detect the activation/repression activity of BLH6a. Yeast with *BLH6a* overexpression could not grow on the medium lacking histidine (Figure 3B), showing that BLH6a could not activate *HIS3* expression. Compared to the VP16 alone, BLH16a:VP16 fusion disturbed the activation of an α-galactosidase gene (Figure 3B), indicating that BLH6a is a transcriptional repressor, not an activator.

We further used ChIP assays in both xylem and leaf protoplasts to examine the interaction of BLH6a and *CAld5H2* promoters. In comparison with a negative control of overexpression of FLAG alone, the *CAld5H2* promoter region was significantly enriched in the immunoprecipitation in both xylem and leaf protoplasts overexpressing FLAG:BLH6a fusion (Figure 3C), confirming the direct binding of BLH6a to *CAld5H2* promoter.

### *BLH6a* Was Co-expressed With *CAld5H* Genes in *P. alba* × *P. glandulosa* Differentiating Xylem

**Figure 4:**
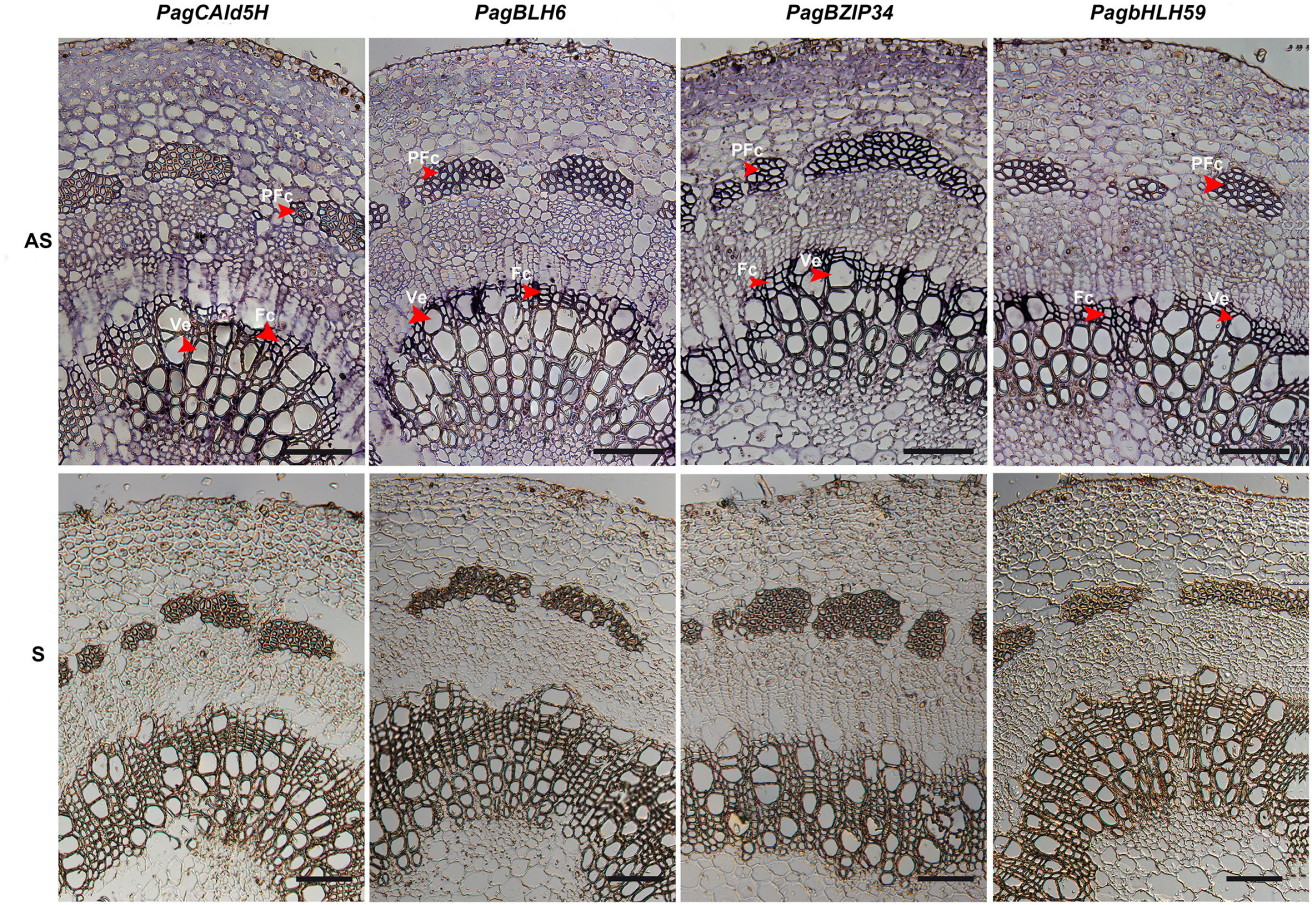
RNA *in situ* localization of *CAld5H, BLH6, BZIP34*, and *bHLH59* in the 8–9 internode stems of *P. alba* × *P. glandulosa stems*. Bars = 200 μm. Arrows show the signals of the vessels (Ve), fiber cells (Fc), and phloem fiber cell (PFc) in the differentiating xylem where signals of RNA *in situ* hybridization were intensified. AS and S represent the sections hybridized with antisense and sense probes, respectively.

The proposed interaction of BHL6a with the *CAld5H2* promoter requires that both genes are expressed in the same cells. We used RNA *in situ* hybridization (RISH) to examine the co-expression of *BLH6a* with *CAld5H* genes in cells of differentiating xylem. *BLH6a* and *BLH6b* are a paralogous gene pair, with 90% nucleotide identity; thus, it is difficult to distinguish transcripts of the two genes using an RNA probe. Similarly, RNA probes for *CAld5H* RISH could not distinguish *CAld5H1* and *CAld5H2*. The RISH detected *CAld5H1/2* transcripts in cross-sections of differentiating xylem, including vessels and fiber cells (Figure 4), consistent with the function of *CAld5H* in monolignol biosynthesis during the SCW biosynthesis. *BLH6a/b* transcripts were also detected in both vessels and fiber cells, in the early stages of the xylem differentiation (Figure 4). We also selected two TFs (bZIP34 and bHLH59), which did not specifically bind the *CAldH2* promoter, for RISH. These two genes showed the same expression pattern as *CAld5H1*/*2* (Figure 4). The RISH showed that *bZIP34* and *bHLH59* were co-expressed with *CAld5H1/2* in all cell types of differentiating xylem, and *BLH6a/b* were co-expressed with *CAld5H1/2* in the early stage of the xylem cell differentiation.

### Protein–Protein Interactions Among 12 TF Candidates

**Figure 5:**
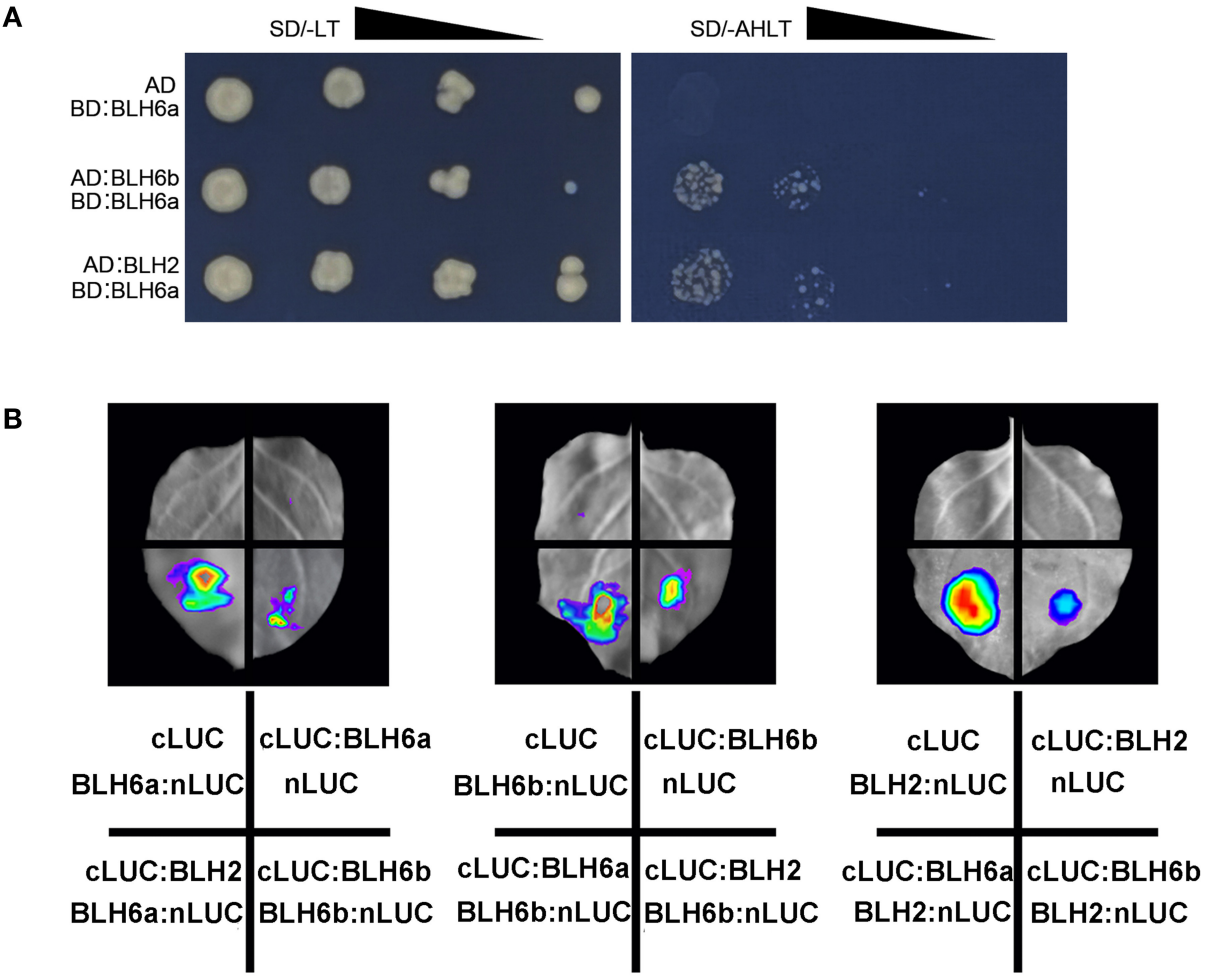
Protein–protein interactions of BLH6a, BLH6b, and BLH2. **(A)** Yeast two hybridizations. BLH6a was fused with Gal4-binding domain (BD). BLH6b and BLH2 were fused with activation domain (AD). The resulting AD constructs (AD:BLH6b, AD:BLH2) and BD construct (BD:BLH6a) were co-transfected into a yeast cell, grown on the SD/-Leu-Trp medium (SD/-LT), and selected on the SD/-Ade-His-Leu-Trp medium (SD/-AHLT). **(B)** Luciferase complementation imaging (LCI) assays for the detection of the interactions between two proteins of BLH6a, BLH6b, and BLH, with one of them being fused to the N-terminal portion of LUC (nLUC), and the other being fused to the C-terminal portion of LUC (cLUC).

All the identified 12 TFs could bind the 556 bp promoter region (fragments 6–7) of *CAld5H2*. It would be interesting to know how they cooperatively regulate *CAld5H2*. We first examined the interactions of BLH6a with other two *CAld5H2*-specific TFs BLH6b and BLH2 by Y2H, and the assays showed that BLH6a could form a dimer with BLH6b and BLH2 (Figure 5A). We further conducted firefly LCI assays for the three BLH members in the *N. benthamiana* leaves (Figure 5B). A strong LUC signal was detected when *BLH6a-nLUC* and *cLUC-BLH2* were co-infiltrated, and reciprocal co-infiltration of *BLH2-nLUC* and *cLUC-BLH6a* also had strong luminescence. Similarly, when each pair of *BLH6a-nLUC/cLUC-BLH6b, BLH6b-nLUC/-cLUC-BLH6a, BLH6b-nLUC/cLUC-BLH2*, and *BLH2-nLUC/cLUC-BLH6b* was co-infiltrated into tobacco leaves, the LUC luminescence was observed. However, no LUC luminescence was observed in negative controls (*cLUC/BLH6a-nLUC, cLUC-BLH6a/nLUC, cLUC/BLH6b-nLUC, cLUC-BLH6b/nLUC, cLUC/BLH2-nLUC*, and *cLUC-BLH2/nLUC*). The LCI assays for the interaction among BLH6a, BLH6b, and bHLH2 were repeated four times. All had positive luminescence, showing that these three proteins could interact with each other.

We further used the LCI assays to examine the remaining possible interactions between any two TFs among 12 TFs. For each two TFs, we examined their interactions reciprocally (i.e., TF1:nLUC/TF2:cLUC and TF2:nLUC/TF1:cLUC), and each assay was conducted twice. Theoretically, 66 interactions could exist among 12 TFs for the heterodimer formation. Our LCI assay identified 58 interactions (Supplementary Figure 3, Supplementary Table 3). Among 58 interactions, 52 were detected for both combinations (TF1:nLUC/TF2:cLUC and TF2:nLUC/TF1:cLUC), and six interactions were detected for one combination (either TF1:nLUC/TF2:cLUC or TF2:nLUC/TF1:cLUC). Except for MYB85b, all other 11 TFs could form homodimers (Supplementary Figure 3).

### Dominant Repression by *BLH6a* in *P. alba* × *P. glandulosa* Inhibited *CAld5H* Gene Expression and Affected SCW Biosynthesis in Differentiating Xylem

**Figure 6:**
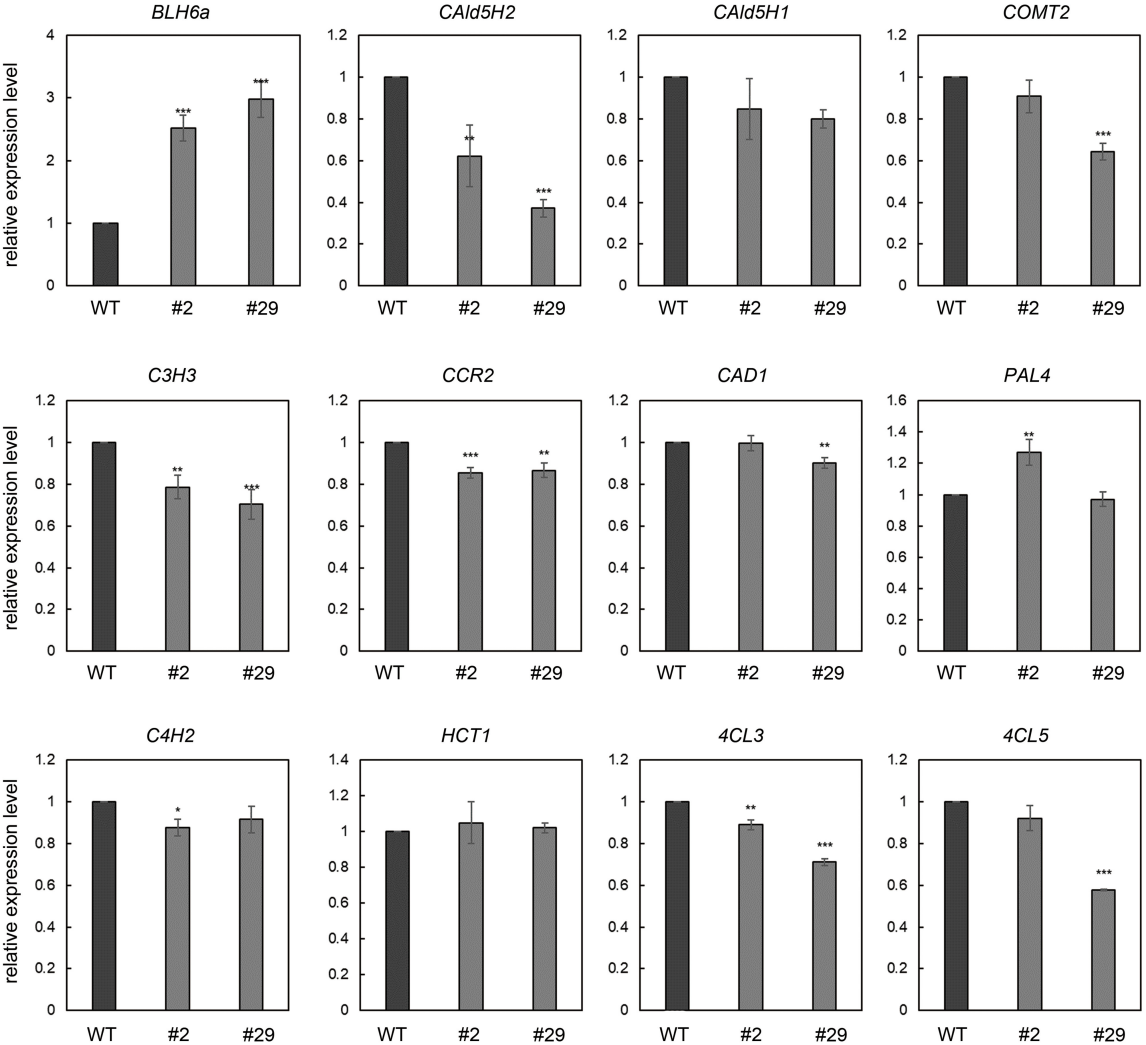
qRT-PCR analysis of monolignol pathway genes in the differentiating xylem of *BLH6a:SRDX* overexpression transgenic *P. alba* × *P. glandulosa*. The transcript abundance in wild type (WT) and transgenic lines 2 and 29 was determined for transgene *BLH6a:SRDX* and monolignol pathway genes *CAld5H2, CAld5H1, COMT2, CAD1, C3H3, CCR2, C4H2, 4CL3, 4CL5, PAL4*, and *HCT1*. Asterisks highlight significant differences by the Student's *t*-test: *p < 0.5, **p < 0.01; ***p < 0.001.

**Figure 7:**
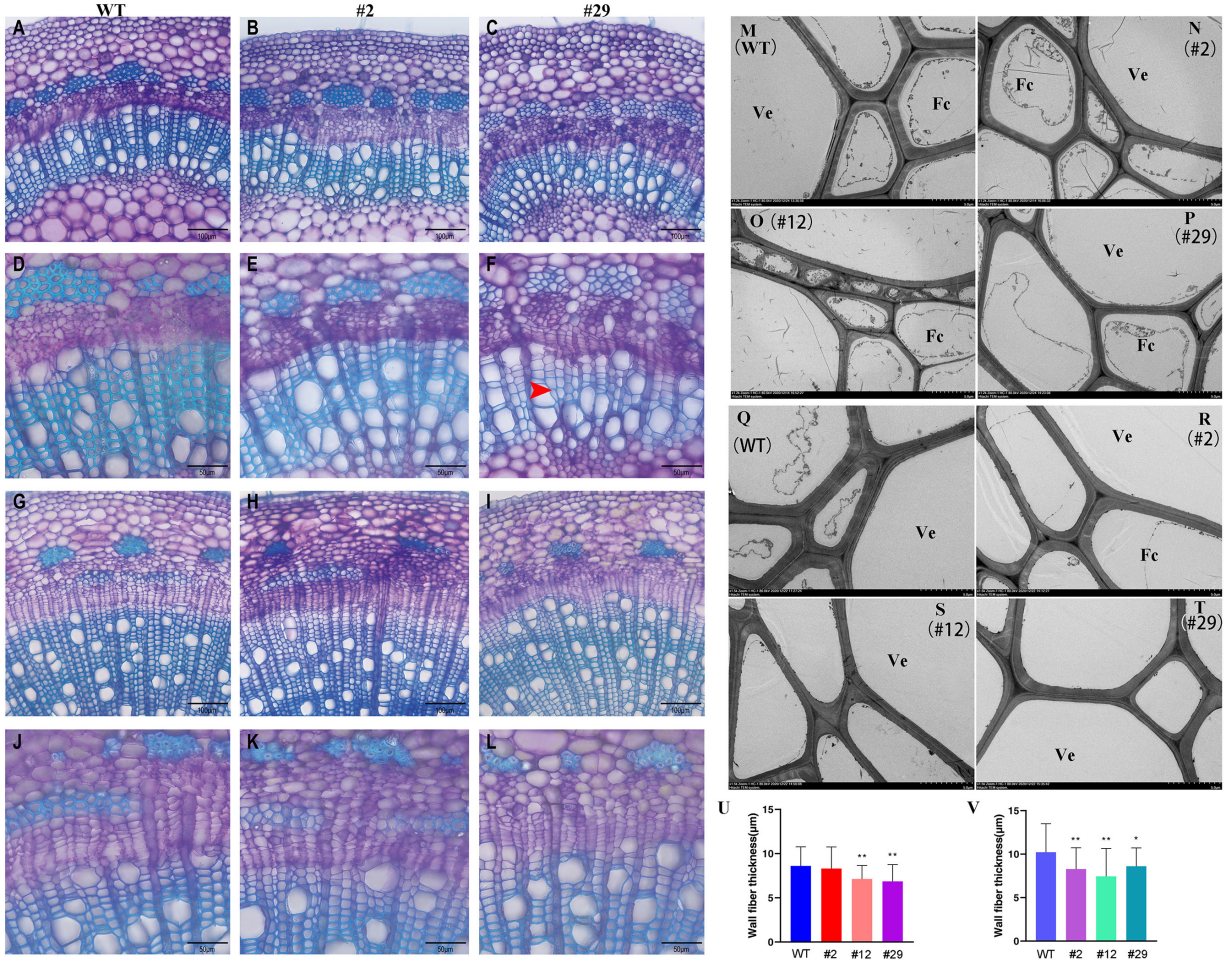
Comparison of xylem structure between *P. alba* × *P. glandulosa BLH6a* dominant repression transgenics and WT. **(A–L)** Light microscopy. **(A–C)** Cross sections of the seventh internode from WT **(A)**, transgenic lines 2 **(B)**, and 29 **(C)** at 200 magnification. **(D–F)** Cross sections of the seventh internode from WT **(D)**, transgenic lines 2 **(E)**, and 29 **(F)** at 400 magnification. **(G–I)** Cross sections of the 14th internode from WT **(G)**, transgenic lines 2 **(H)**, and 29 **(I)** at 200 magnification. **(J–L)** Cross sections of the 14th internode from WT **(J)**, transgenic lines 2 **(K)**, and 29 **(L)** at ×400 magnification. Bars = 100 μm in **(A–C**, **G–I)** and 50 μm in **(D–F**, **J–L)**. The red arrow shows the thinner cell wall. **(M–T)** Transmission electron microscopy. **(M–P)** Cross-sections of the eighth internode from WT **(M)**, transgenic lines 2 **(N)**, 12 **(O)**, and 29 **(P)**. **(Q–T)** Cross-sections of the 14th internode from WT **(Q)**, transgenic lines 2 **(R)**, 12 **(S)**, and 29 **(T)**. Bars = 5 μm. Ve, Vessel; Fc, Fiber cell. **(U,V)** Statistics of fiber cell wall thickness in the eighth internode **(U)** and the 14th internode **(V)** of WT, transgenic lines 2, 12, and 29. *p < 0.05, **p < 0.01, determined by the Student's *t-*test.

*Arabidopsis* BLH6 was identified as a transcriptional repressor of REVOLUTA during the SCW formation (Liu et al., 2014). Our results showed that BLH6a is a transcriptional repressor of *CAld5H2* in poplar. We identified 12 upstream TFs of CAld5H2, indicating a possibility of redundancy of these TFs. To avoid the redundancy, we exploited the Chimeric REpressor gene Silencing Technology (CRES-T) (Mitsuda et al., 2011) to investigate the regulation of BLH6a on CAld5H2 *in vivo*. We obtained 36 transgenic *P. alba* × *P. glandulosa* lines for overexpression of *BLH6a:SRDX*, which encoded a chimeric repressor generated by the fusion of the BLH6a protein with a plant-specific SRDX. All transgenic lines grew as normal as wild type (WT). After the quantitative reverse transcription (qRT)-PCR analysis of transgene (*BLH6a:SRDX*) in the xylem of 36 transgenic lines (Supplementary Figure 4), we selected two lines (#2 and #29) to measure the expression levels of *CAld5H2* and *CAld5H1*, and nine other genes encoding the enzymes of monolignol pathway, including *PAL4, CAD1, COMT2, C3H3, C4H2, CCR2, 4CL3, 4CL5*, and *HCT1*. Compared to WT, *CAld5H2* transcript levels were significantly reduced in both lines 2 and 29. The reduction in transcript abundance of *CAld5H2* in line 29 (62.9%) is greater than line 2 (37.7%), in accordance with the higher transgene expression level in line 29 (Figure 6). The transcript abundance of *CAld5H1* in lines 2 and 29 was not changed significantly. Transcript reduction was also observed for *COMT2, CAD1, C3H3, CCR2, 4CL3*, and *4CL5*, but the extent of reduction is much less than that of *CAld5H2* (Figure 7). The qRT-PCR results indicate that BLH6a may interact more specifically with the *CAld5H2* promoter, consistent with the results of Y1H assays (Figure 2).

To study the effects of dominant repression of BLH6a regulation on the wood formation, we used lines 2 and 29 to examine the cell wall structure at the seventh and the 14th stem internodes using light microscopy. Line 29 had a higher level of *BLH6a:SRDX* overexpression than line 2. Accordingly, it was observed that the cell wall thickness of vessels and fiber cells in both the seventh and the 14th internodes of line 29 was decreased (Figures 7A–L). We further used transmission electron miscopy to compare the xylem cell wall thickness between transgenic lines (#2, #12, and #29) and WT. Transgenic line 12 had a similar expression level of *BLH6a:SRDX* as line 29 (Supplementary Figure 4). The measurement of 50 fiber cells showed that the cell wall thickness was significant decreased in both the eighth and the 14th internodes of lines 12 and 29 (Figures 7M–V), indicating that the overexpression of *BLH6a:SRDX* affected the SCW formation in the differentiating xylem.

To investigate effects on lignin biosynthesis by the *BLH6a:SRDX* overexpression, we determined lignin content and composition in transgenic lines 2 and 29 (Table 1). We only observed a slight and significant decrease of cellulose content in line 29. Compared to WT, the contents of lignin and the three monomers were not changed significantly, showing that lignin biosynthesis is not affected significantly in the transgenic wood.

**Table 1:** Lignin content and compositions in the *BLH6a:SRDX* overexpression transgenics.

**Line**	**Cellulose content**	**Hemicellulose content**	**Acid-insoluable lignin (%CWR)**	**Acid-soluable lignin (%CWR)**	**H** **(μmol/g CWR)**	**G** **(μmol/g CWR)**	**S** **(μmol/g CWR)**
WT	48.53 ± 0.37	16.67 ± 0.34	19.88 ± 0.26	6.34 ± 0.17	2.86 ± 0.11	226.86 ± 0.22	488.83 ± 10.69
#2	47.10 ± 0.29	17.18 ± 0.45	19.24 ± 0.10	6.76 ± 0.01	2.60 ± 0.19	221.44 ± 4.71	547.59 ± 12.91
#29	46.21 ± 0.11^*^	16.73 ± 0.26	20.37 ± 0.69	6.45 ± 0.12	2.75 ± 0.01	241.82 ± 7.17	532.77 ± 9.68

*Values are means ± SE. SEs represent two biological replicates. WT, wild type; #2, BLH6a:SRDX overexpression transgenic poplar line 2; #29, transgenic line 29. The asterisk represents the significant increase of cellulose content in #29 by the Student's t-test (*

* *p < 0.05)*.

## Discussion

Wood is the major resource for timber, paper, and pulping. Because wood is composed of SCW in vascular plants, extensive studies have been carried out to understand the composition, function, and biosynthesis of the SCW, particularly in the model plant *Arabidopsis thaliana* and several species of poplar. In these model systems, many TFs, including NACs and MYBs, were identified as key regulators during the SCW formation (Zhong et al., 2008; Ye and Zhong, 2015). Hierarchical gene regulatory networks (hGRNs) controlling SCW thickening are under construction using many different bioinformatic and biochemical strategies and techniques. One recent combination of methods used top-down Gaussian Graphical Modeling (GGM) to infer correlated molecular interactions and the direction of the correlated effects (Lin et al., 2013; Chen et al., 2019). Network construction uses relative abundance based on the global transcript sequencing (RNA-seq), chromatin binding to construct a four-layered hGRN directed by PtrSND1-B1, a master regulatory NAC protein affecting wood formation (Lin et al., 2013; Chen et al., 2019). Alternatively, bottom-up GGM was used to build a two-layered hGRN for laccases, which polymerize lignin monomers (Lu et al., 2013). Using enhanced Y1H to identify protein**–**DNA interactions, networks controlling the vascular cambium development and SCW formation have been constructed (Taylor-Teeples et al., 2015; Xu et al., 2016; Yeh et al., 2019; Smit et al., 2020). Through transcriptional network construction, additional interactions and feedback, and feed-forward regulations, have been described (Ko et al., 2014; Smit et al., 2020). In this study, we constructed a TF-prey library containing 227 xylem-specific TFs and conducted Y1H assays to identify TFs regulating *CAld5H2* in hybrid poplar. We identified 12 TF candidates and focused on the characterization of three BLH family proteins. BLH proteins belong to the plant-specific three-amino acid loop extension (TALE) superclass of the homeodomain protein family. In *Arabidopsis*, BLH6a has been indicated to regulate SCW formation through interacting with KNOTTED ARABIDOPSIS THALIANA7 (KNAT7) (Liu et al., 2014), and BLH2 has been indicated to regulate demethylesterification of homogalacturonan in seed mucilage (Xu et al., 2020). In the *Arabidopsis blh6* mutant, expressions of some of CELLULOSE SYNTHASE (CESA) and lignin pathway genes, such as *CesA7, CesA8*, and *F5H*, were increased (Liu et al., 2014). Our results confirmed the regulation of BLH6a on *CAld5H2* in poplar by transient overexpression, stable dominate repression, and ChIP–qPCR. Our analyses on the activation/repression ability of poplar BLH6a are consistent with the conclusion that *Arabidopsis* BLH6 is a transcriptional repressor (Liu et al., 2014). Because BLH6a is a transcriptional repressor, overexpression of *BLH6a:SRDX* would enhance its repression ability, which will cause a similar phenotype of overexpression transgenics. Consistently, decreased cell wall thickness is observed in *Arabidopsis* overexpressing a *BLH6* gene (Liu et al., 2014).

Different methods for the hGRN construction and TF identification have their advantages and limitations. Each method may generate false positives. For network prediction by GGM algorithms, more samples with more variation of the gene expression levels improve the precision of the network. The constructed networks and TFs included in the network by different strategies are not the same. For example, NAC123 directly targets *CCoAOMT1* in *P. trichocarpa* by ChIP–qPCR in xylem protoplasts (Chen et al., 2019). Our Y1H showed that NAC123 (also named SND2 in this study) could bind the promoters of *CAld5H2, COMT2, PAL4, C4H2*, and *4CL5*. In the *P. trichocarpa* network (Chen et al., 2019), the TF WBLH2 interacts with *CAld5H1* and *HCT1* promoters, whereas in this study its homolog BLH2 from *P. alba* × *P. glandulosa* bound *CAld5H2* and *CAld5H1* promoters and did not bind the promoters of other monolignol pathway genes. Further experiments, such as ChIP assays in xylem cells (not xylem protoplasts) and electrophoretic mobility shift assays (EMSA) are needed to verify these interactions. The protein**–**DNA interactions ought to be proven before carrying out experiments to show that they are expressed in the same cells. Based on transcriptome data from a series of xylem cryosections (Sundell et al., 2017), our 12 TF genes are co-expressed with *CAld5H2* (Supplementary Figure 1). The RISH has provided evidence of co-expression of *BLH6a/b, BZIP34*, and *bHLH59* with *CAld5H1/2*. Further experiments, laser capture microdissection, or single-cell RNA sequencing (scRNA-seq) may provide additional information on gene co-expression.

Note that the networks constructed in different species may not be the same. In *Arabidopsis*, MYB46/83, which is located in the second layer directly regulated by SND1, can directly regulate the bottom layer SCW biosynthetic genes, including three CesA encoding genes (*CesA4, 7*, and *8*), seven monolignol pathway genes (*PAL1, 2, 4, 4CL3, CCR, CCoAOMT1*, and *CAD*) and four xylan biosynthetic pathway enzyme encoding genes (*FRA8, IRX8, IRX9*, and *IRX14*) (Zhong and Ye, 2012; Kim et al., 2013; Ko et al., 2014). However, in *P. trichocarpa*, using ChIP assays in xylem protoplasts overexpressing *PtrMYB021* (homolog of MYB46) only identifies four bottom layer genes (*PtrIRX9, PtrIRX14-L, PtrFRA-1*, and *PtrPAL2*) as the direct targets of PtrMYB021 (Chen et al., 2019). Further ChIP assays on the five TFs (targets of PtrMYB021) in the third layer, including PtrMYB090, PtrMYB161, PtrMYB174, PtrNAC123, PrWBLH1, and PtrWBLH2, identified three *CesA* gene promoters and 10 monolignol pathway gene promoters (Chen et al., 2019).

The poplar genome has had a historical whole-genome duplication event, generating about 8,000 pairs of duplicated genes (Tuskan et al., 2006). During evolution, both promoter and gene body between the gene pair could have diverged. In *Arabidopsis*, MYB85 overexpression induces *4CL* expression and causes ectopic lignin deposition (Zhong et al., 2008). In our study, the identified 12 TFs contain two poplar MYB85 paralogs (MYB85a and MYB85b). Y1H assays showed that MYB85a only interacted with *CAld5H2* and *COMT2* promoters, whereas MYB85b interacted with *CAdl5H2, COMT2, C3H3, CAD1, PAL4*, and *4CL3* promoters, showing the different abilities of these two MYB85 members in activating monolignol biosynthesis. In *P. trichocarpa*, the two homologs 4CL3 and 4CL5 have different regulatory metabolic activity, indicating that the difference in promotor binding of *4CL3* and *4CL5* may be related to their different metabolic roles (Chen et al., 2013). All 12 TFs could bind the *CAld5H2* promoter, but only three TFs could bind the *CAld5H1* promoter, indicating promoter divergence.

Many studies have shown that one gene may be regulated by multiple TFs, and multiple TFs binding to the same promoter could regulate target genes by forming protein complexes (Chen et al., 2019). Pairwise interactions among three TFs, PtrMYB090, PtrMYB161, and PtrWBLH1 have been detected in *P. trichocarpa*, and these three TFs may form a ternary complex regulating *CAld5H*. Our LCI assays for pairwise interactions among 12 TFs identified extensive interactions between TFs, indicating many TFs may form many dimers. In the *BLH6a:SRDX* overexpression transgenics, the *CAld5H2* gene was downregulated up to 62%, but the *CAld5H1* gene was not downregulated significantly. S lignin content was not changed in the *BLH6a:SRDX* overexpression transgenic wood, which could be due to the redundancy between *CAld5H1* and *CAld5H2*. Thus, it is necessary to perform Y1H to identify the TFs that specifically bind to the *CAld5H1* promoter. Although the CRES-T technology could avoid redundancy, using the SRDX system for a transcriptional repressor may not make much difference in some cases, which may be another possible reason for the unchanged S lignin content. When the TF of interest is a transcriptional repressor, transgenic plants expressing a chimeric repressor exhibit an enhanced phenotype (Mitsuda et al., 2011). Further using the CRISPR-CAS9 system to knock out the candidate TF genes regulating *CAld5H1/2* in poplar is needed to understand their roles in lignin biosynthesis. Although lignin content and composition did not show alteration in *BLH6a:SRDX* overexpression transgenic wood compared to WT, we observed that the xylem cell wall thickness was decreased significantly. This phenotype may be caused by the interactions of BLH6a with other TFs. The conserved BELL and SKY domains in BLH proteins comprise a conserved bipartite MID domain, which can interact with the KNAT MEINOX domain to form heterodimers (Bellaoui et al., 2001), and the decrease is likely associated with the repression of *REVOLUTA* by BLH6 and KNT7 interactions. OVATE FAMILY PROTEIN1 (OFP1) and OFP4 are components of the BLH6-KNAT7 multi-protein complex and may modulate the activity of the BLH6-KNAT7 complex (Liu and Douglas, 2015). Further investigations are needed to study the heterodimer formation among the identified TFs for efficient regulation of *CAld5H* promoter activity.

Multiple TFs may have the combinatorial coordination to activate or suppress target gene expression. Transcriptional activation and repression strength and sequence specificity of TFs depend on their expression levels. The RISH showed *BLH6a* transcript abundance in the vessels and fiber cells at the early stages of xylem differentiation. Two other genes *BZIP34* and *bHLH59*, which were expressed throughout the xylem, have the same expression profile with *CAld5H1/2*. These 12 genes may function at different locations, either activating or suppressing *CAld5H* genes. Different cells may have different proportions of different protein**–**protein interactions, thus affecting *CAld5H* gene expression through different protein complex composition and abundance. Further investigation is needed to determine whether these TFs are activators or repressors and to study their potential for redundant and combinatorial regulation.

## Conclusion

We identified 12 upstream candidates of *CAld5H2*. The regulation of one TF, BLH6a, on *CAld5H2* was substantiated by transient effector–reporter assays, dominant repression, and ChIP–qPCR. Further understanding the TFs regulating *CAld5H2* and their mechanisms of action will lead to novel strategies for engineering S subunit levels in lignin. Such information will be important for the development of S subunits in gymnosperm wood, which are currently absent in gymnosperms.

## Data Availability Statement

The original contributions presented in the study are included in the article/Supplementary Material, further inquiries can be directed to the corresponding author/s.

## Author Contributions

QL designed and supervised the project. QW, XD, HP, YC, XH, HL, and XY performed the experiments. FL, HW, RS, and QL analyzed the data. QW, HW, RS, and QL wrote the manuscript. All authors contributed to the article and approved the submitted version.

## Conflict of Interest

The authors declare that the research was conducted in the absence of any commercial or financial relationships that could be construed as a potential conflict of interest.

## Supplementary Material

Supplementary Figure 1 Co-expression analysis of 12 TF genes with *CAld5H1* and *CAld5H2*.

Supplementary Figure 2 Expression patterns of 12 TFs and two *CAld5H* genes in stem cryosections of *P. tremula*.

Supplementary Figure 3 Protein**–**protein interactions among 12 TFs by LCI assays.

Supplementary Figure 4 qRT-PCR of transgene *BLH6a:SRDX* in the leaves of 36 transgenic lines.

Supplementary Table 1 Primers used in this study.

Supplementary Table 2 12 TF candidates regulating *CAld5H2*.

Supplementary Table 3 Summary of interactions among 12 TFs.

Supplementary Data Sheet 1 227 xylem-specific TFs in *Populus trichocarpa*.

Supplementary Data Sheet 2 Sequence motif analysis of the fragments 6 and 7 in *CAld5H2* promoter.
