# Corrigendum: BEL1-like Homeodomain Protein BLH6a Is a Negative Regulator of *CAld5H2* in Sinapyl Alcohol Monolignol Biosynthesis in Poplar

**DOI:** 10.3389/fpls.2021.761291

**Published:** 2021-09-09

**Authors:** Qiao Wang, Xinren Dai, Hongying Pang, Yanxia Cheng, Xiong Huang, Hui Li, Xiaojing Yan, Fachuang Lu, Hairong Wei, Ronald R. Sederoff, Quanzi Li

**Affiliations:** ^1^State Key Laboratory of Tree Genetics and Breeding, Chinese Academy of Forestry, Beijing, China; ^2^Research Institute of Forestry, Chinese Academy of Forestry, Beijing, China; ^3^Department of Energy Great Lakes Bioenergy Research Center, Wisconsin Energy Institute, Madison, WI, United States; ^4^College of Forest Resources and Environmental Science, Michigan Technological University, Houghton, MI, United States; ^5^Forest Biotechnology Group, Department of Forestry and Environmental Resources, North Carolina State University, Raleigh, NC, United States

**Keywords:** lignin, *CAld5H2*, BEL1-like homeodomain protein, transcription factor, yeast one hybrid

When originally published, the article title contained a typographical error. The correct gene name should be “*CAld5H2*” instead of “*CAl5H2*” as originally published. The correct title is “BEL1-like Homeodomain Protein BLH6a is a Negative Regulator of *CAld5H2* in Sinapyl Alcohol Monolignol Biosynthesis in Poplar.”

The authors state that this does not change the scientific conclusions of the article in any way. The original article has been updated.

## Publisher's Note

All claims expressed in this article are solely those of the authors and do not necessarily represent those of their affiliated organizations, or those of the publisher, the editors and the reviewers. Any product that may be evaluated in this article, or claim that may be made by its manufacturer, is not guaranteed or endorsed by the publisher.

