# Supplementary material for: BEL1-like Homeodomain Protein BLH6a Is a Negative Regulator of CAld5H2 in Sinapyl Alcohol Monolignol Biosynthesis in Poplar
Source: Front Plant Sci. 2021 Jun 25;12:695223. doi: 10.3389/fpls.2021.695223 (PMC8269948; doi:10.3389/fpls.2021.695223)
Supplement: Supplementary Figure 1 — Co-expression analysis of 12 TF genes with CAld5H1 and CAld5H2. [file Data_Sheet_3.docx]

**Fig. S1:** Coexpression analysis of 12 TF genes with *CAld5H1* and *CAld5H2.* The network is generated using the AspWood web resource (<http://aspwood.popgenie.org>).


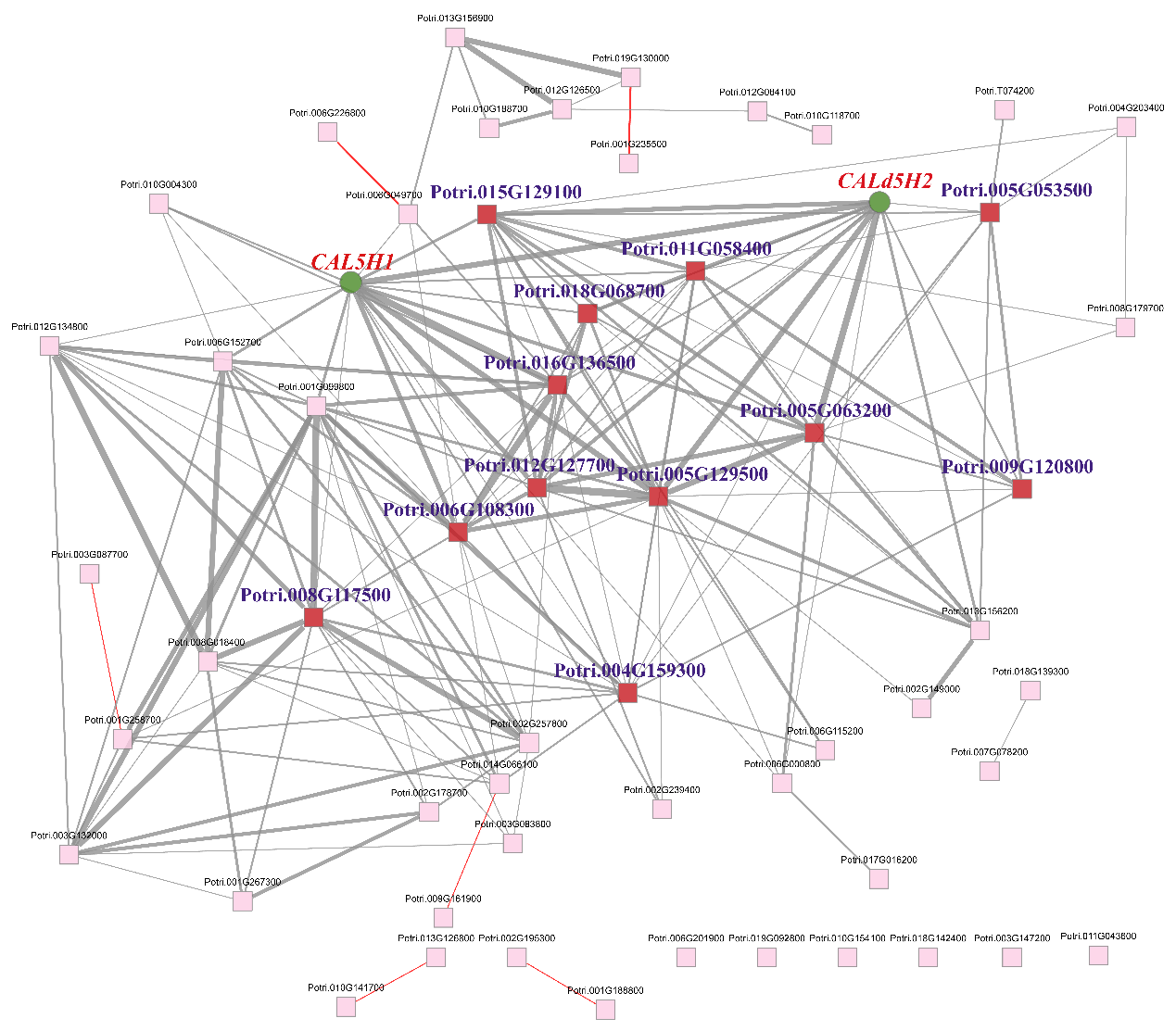


**Fig. S2:** Expression patterns of 12 TF and two *CAld5H* genes in stem cryosections of *P. tremula*, generated using the AspWood web resource (<http://aspwood.popgenie.org>).


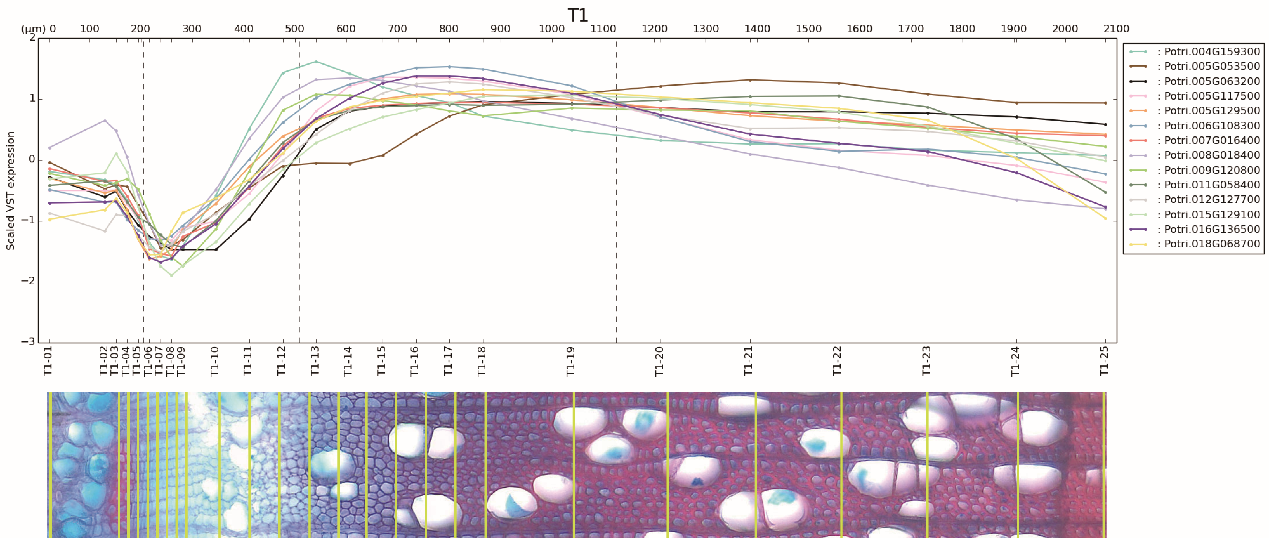


**Fig. S3:** Protein-protein interactions among 12 TFs by Luciferase Complemntary Image (LCI) assays. Two proteins were co-expressed in tobacco leaves, with one fused to N-ermianl portion of LUC (nLUC) and the other fused to C-termianl portion of LUC (cLUC). The 12 TFs are listed in Supplementary Table S2, and the numbers of interactions for homodimer and heterodimer formation among 12 TFs are shown in Supplementary Table S3.

*
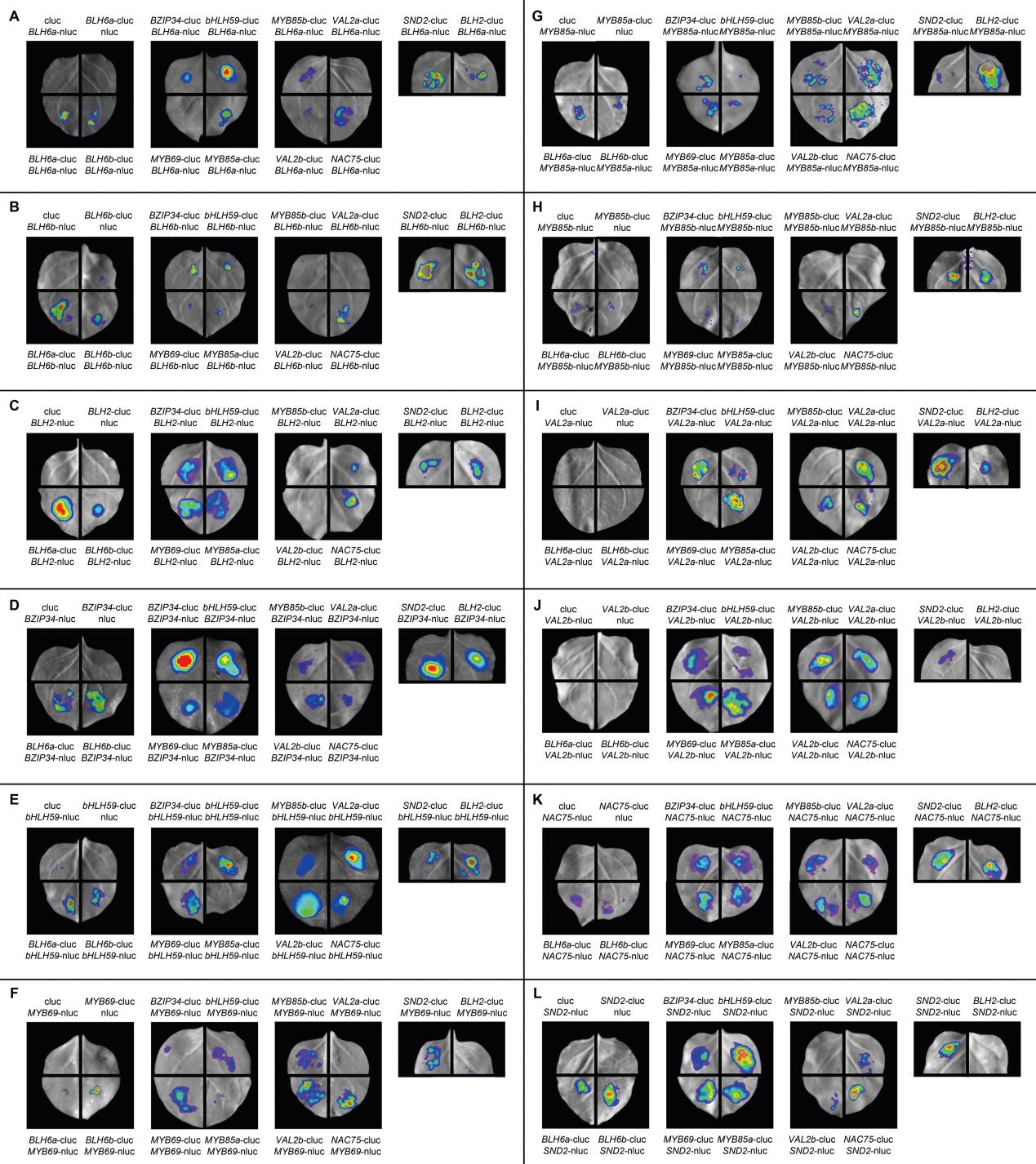
*

**Fig. S4:** Detection of transgene *BLH6a:SRDX* expression levels by qRT-PCR in the leaves of 36 transgenic lines.

*
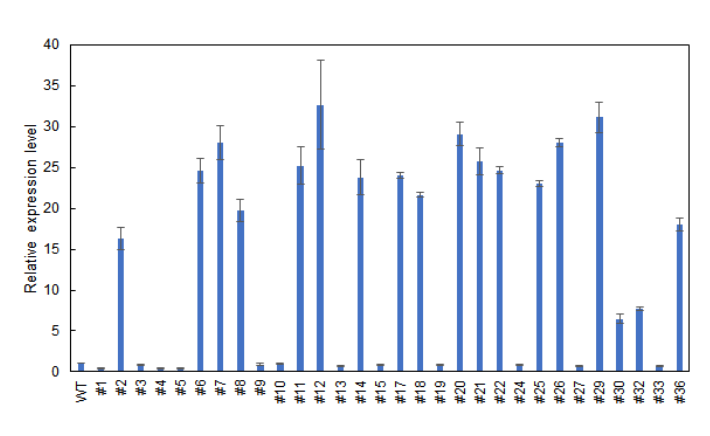
*
