# Supplementary material for: BEL1-like Homeodomain Protein BLH6a Is a Negative Regulator of CAld5H2 in Sinapyl Alcohol Monolignol Biosynthesis in Poplar
Source: Front Plant Sci. 2021 Jun 25;12:695223. doi: 10.3389/fpls.2021.695223 (PMC8269948; doi:10.3389/fpls.2021.695223)
Supplement: Supplementary Table 1 — Primers used in this study. [file Table_1.docx]

**Supplementary Table S1** Primers used in this study

| **Primers for 227 xylem-specific TF amplification from *P. trichocarpa* for Y1H (Restriction enzyme sites are underlined)** | |
| --- | --- |
|  |  |
| 1F | GGAATTCCATATGATGATGGCAATGTCCTGCAAGG |
| 1R’ | CGCGGATCCTTCCACAGCAGAGTTGCAAAAGA |
| 2F | CCGGAATTCATGGCAGCCTTTGCAGGAAC |
| 2R | CGCGGATCCTGATTCAGCAGCAATTTCTACAGC |
| 3F | CCGGAATTCATGGAGGAGGGGAGGTTTCA |
| 3R | CGCGGATCCTCCAAACAAGTCATATTGCTCCAT |
| 4F | GGAATTCCATATGATGAATAGAGGGGTTTTGCAGAG |
| 4R | CCGGAATTCTCGGAACCCCCCTCCAAGAG |
| 5F | CCGGAATTCATGGAAACCTCCTCCCCTTCC |
| 5R’ | CGCGGATCCGTTGTTTCCACAGCAGCTTCCT |
| 6F | CCGGAATTCATGACTTGGTGCAATGACTGC |
| 6R | CGCGGATCCGGGGATAAAAGAAGATCCGTCA |
| 7F | TCCCCCGGGTATGGCTGGGATTGATGATAATGTTG |
| 7R’ | atgCGAGCTCGTTCACATGGCTGCTATTGCGA |
| 8F | TCCCCCGGGTATGGAAATGTCCTGCAAGGATG |
| 8R | CGCGGATCCGACAAAAGACCAGTTTATAAACATAAAGCA |
| 9F | CCGGAATTCATGGCGCTTTCTATACACAGTAAGG |
| 9R’ | ATGCGAGCTCACCATCATCTGTGAACCCATTCA |
| 10F | TCCCCCGGGtATGATAGATCTTAACACAACTGAAGAAGA |
| 10R’ | CGCGGATCCGAGCCTTGTTAGCATTTGGAAT |
| 11F | CGCGGATCCATATGGGTCACCATTCTTGCTGC |
| 11R | atCCGCTCGAGTAGAGATGAAGGGAAAGAAGAGAG |
| 12F | CGCGGATCCatATGGGCAGACAACCTTGTTGC |
| 12R | atCCGCTCGAGATGCTTGCCACCCATGTCTAG |
| 13F | CGCGGATCCatATGACTTGGTGCAATGACTGC |
| 13R | atCCGCTCGAGAGGGATAAGAGAAGATCCATCATG |
| 14F | CCGGAATTCATGCAAGAACCAAACTTGGGC |
| 14R | CGCGGATCCCCTTTTGCGCTTGGACTTCAA |
| 15F | CGCGGATCCatATGGGTCACCATTCTTGCTGC |
| 15R | atCCGCTCGAGTAGAGATGAAGGGAAAGAAGAGAG |
| 16F | CCGGAATTCATGCCTGAAGATATGGTGAATCTATC |
| 16R | CGCGGATCCTACCGACAAGTGGCATAATGGG |
| 17F | CGCGGATCCatATGGCTACCTATTACACTAGTTCGA |
| 17R | atCCGCTCGAGAGCAACGAAATCATGAAATACATGGG |
| 18F | CCGGAATTCATGGATAAAGAAACTAACCGAGAAAGC |
| 18R | CGCGGATCCAGTTTTTCCAATTCTCAAGGACGA |
| 19F | CGCGGATCCatATGGGAATAGCTACAACACCTCC |
| 19R’ | atCCGCTCGAGTGGCTTGCTTCCTTTCTCTATCT |
| 20F | CCGGAATTCATGCCTGAGGATATGATGAATCTATC |
| 20R | CGCGGATCCTACCGATAAGTGGCATAATGGGTC |
| 21F | CCGGAATTCATGGAGATAACACAACCACCAC |
| 21R | CGCGGATCCACGAGGACCCAGTAGTAACCT |
| 22F | TCCCCCGGGtATGGCTACCTATTATACTAGTTCAAATAATCAA |
| 22R’ | atCCGCTCGAGAGAGGTTCCCATATCACCTTTT |
| 23F | CCGGAATTCATGGAGAAAACAGAATCACCACC |
| 23R | CGCGGATCCACGAGGACCCAGCAGTAACC |
| 24F | CCGGAATTCATGGCGCTTTCTATGCACAGT |
| 24R’ | CGCGGATCCAAGCAGGAGGAACATTCTGCA |
| 25F | CCGGAATTCATGAAAAATCTTGACAAGCAGACAAA |
| 25R | CGCGGATCCTTTCTCAAATATGCATATTCCGATATCAC |
| 26F | CCGGAATTCATGGCATTCACGGGAACCCT |
| 26R | atCCGCTCGAGAGATTGCTCTTCTGGCTTTTCC |
| 27F | CCGGAATTCATGGGAAGGCAACCTTGCTG |
| 27R | CGCGGATCCGTGCTTGTGTTCCATCTCTAATGT |
| 28F | CGCGGATCCatATGGGAATAGCTACACCTCCA |
| 28R | taCGCTCGAGACAACCCCCAAAATCTCTAACA |
| 29F | CCGGAATTCATGAAGGAGGAGGCTCAACTA |
| 29R | CGCGGATCCCATGTAATCCATTGCAGGGTTG |
| 30F | CCGGAATTCATGGAAGATCTTAAAGGTAGCTCAAG |
| 30R | CGCGGATCCAGAGATTCCCACACCGAGAA |
| 31F | CCGGAATTCATGGGAAGGCAACCTTGCTG |
| 31R | CGCGGATCCGCGATTGAGTTCCATCTCTAATATGT |
| 32F | CCGGAATTCATGGCCAATAATCCAACCGAA |
| 32R | CGCGGATCCTGATGGGGGATTTGTTTCTGG |
| 33F | CCGGAATTCATGCCAGTTCCTCTTGCACC |
| 33R | CGCGGATCCGGCATTGAACTTCTCTTCAGTGG |
| 34F | CCGGAATTCATGGAGGAGGCTAGTAGTGGT |
| 34R’ | ATGCGAGCTCAGGATTAGCAGCACTGTTCTGA |
| 35F | CCGGAATTCATGGATAGCCAAGATCCCAGAA |
| 35R' | atgCGAGCTCTCGACATTGACAGCAGATTCA |
| 36F | CGCGGATCCatATGATGCCGGCAAAATCTGAA |
| 36R | atCCGCTCGAGGTTCAAGTCTGAAGACAGCATGA |
| 37F | CCGGAATTCATGTGTACTAGAGGCCATTGGAG |
| 37R | atgCGAGCTCAGCTGAGAAAAAATCAATGAATGGAAC |
| 38F | CGCGGATCCatATGGCCTCTGGGTTTTCAGG |
| 38R | atCCGCTCGAGACGCCAAGCAGATGCGACTG |
| 39F | CGCGGATCCatATGGAAAGTGGATTTCCTGATCG |
| 39R’ | ATCCGCTCGAGAAGCGCTGTCCATCCATGTTTAT |
| 40F | TCCCCCGGGtATGTTGGATCTCAATCTTGGTATCAG |
| 40R | CGCGGATCCTCTCTTGTAAGAGTAAAGAGAGGGGT |
| 41F | CCGGAATTCATGAAGGAAGGGATGAGTAAATCTTG |
| 41R | CGCGGATCCCGTCTGCTTTCTCATCTCTAACAC |
| 42F | CCGGAATTCATGGACAACAATACCTCTCGCT |
| 42R | CGCGGATCCTGGACCCGTTAATAATCTTCCC |
| 43F | CCGGAATTCATGCACCAAATAAGCCAACAAGA |
| 43R | atCCGCTCGAGGAAAGAGGAAAAACATTCATGTTCTG |
| 44F | TCCCCCGGGtATGGGGAGGCATTCTTGCTGT |
| 44R | CGCGGATCCAAGGGATTGTCCAAAAGCCAC |
| 45F | CCGGAATTCATGGCGTCCTCGTCCTCGTC |
| 45R’ | CGCGGATCCAGCTCAAAAGCATCTAGGCTATCAA |
| 46F | TCCCCCGGGtATGTCTTTAGTTGGACCTGCAGA |
| 46R | CGCGGATCCCTTCCATGCTGAAGCAACAATCA |
| 47F | CCGGAATTCATGGCTAGCCACAGAGTTGGA |
| 47R’ | CGCGGATCCAAAGTTGGCGAAATGATTCTGTGAAG |
| 48F | TCCCCCGGGtATGGAGAGTGAGTACTTTTTCAATGC |
| 48R | CGCGGATCCGAGCTCGATTTTCATGTGGGA |
| 49F | CCGGAATTCATGGTTAGCTTACGGAGGCG |
| 49R | CGCGGATCCAGAGGCTGATGAGTCTGGTTC |
| 50 | CDS sequence was synthesized by GENERAY company |
| 51F | TCCCCCGGGtATGGCTACCTATTACCCAACTTCA |
| 51R‘ | atCGCTCGAGTGTAGCAGCAGCAGACATAAGATT |
| 52F | CGCGGATCCatATGGGTAGACAACCTTGTTGCG |
| 52R | atCCGCTCGAGACGCTTGCCACCCATGTCTA |
| 53F | CCGGAATTCATGTGTGGACTTAAAGAGGAAGATC |
| 53R | CGCGGATCCTAGAGACCTGTGCTGTTGTTGT |
| 54F | CCGGAATTCATGATTTTATTTTTCTTTTCTTTTTATGTCTTCATC |
| 54R | CGCGGATCCCCATTTTCTCTGAAGCTTTCCTTT |
| 55F | CCGGAATTCATGACTACTAAGAGTAATATGGCTTCCA |
| 55R | atgCGAGCTCGACCAACCCATGATGATCCTG |
| 56F | TCCCCCGGGGATGAGGAAGCCAGAGGCCTC |
| 56R | atgCGAGCTCTTGGAAATCAAGGAATGGAAAGGC |
| 57F | CCGGAATTCATGGATCAAGGAGGAAGAGAAG |
| 57R | atCCGCTCGAGGAAAGAGGTAAAGCATTCATGTTCTG |
| 58F | CCGGAATTCATGAGTACTAGGTGTAGCATGGC |
| 58R | CGCGGATCCCCCATGATGATCCTGGTTGC |
| 59F | CCGGAATTCATGTCAAGGCAATCCCTACTTC |
| 59R | CGCGGATCCGTTAAGATTAAGTTTTGCCATCTGCC |
| 60F | CCGGAATTCATGGGTTATATTTGTGATTTTTGTGGG |
| 60R | CGCGGATCCGAAACTTCTTGTTTGGCTCAGTG |
| 61F | CGCGGATCCatATGATGGCTATGAATAGAGGAGGA |
| 61R’ | atCCGCTCGAGTCTTCTCACCATGTTACAGAACTCA |
| 62F | CGCGGATCCatATGGGGTGCCTTGATGATGG |
| 62R | atCCGCTCGAGACATGCTGCCGAAGGATTAGTG |
| 63F | CCGGAATTCATGTATCAGCTGGAGAGCGTT |
| 63R | atgCGAGCTCAACATCACTGCCTGAAACTTTCT |
| 64F | CCGGAATTCATGGCGTCTTCTTCTGATCCG |
| 64R | CGCGGATCCCTCCTCTGGCCGAAAGAAAC |
| 65F | CCGGAATTCATGGCAGATGACAAGGGTTTATT |
| 65R | CGCGGATCCTTGAGATAGTAAAATGGTTGCACTTTC |
| 66F | CCGGAATTCATGGCAAATGACAAGGGTTTATTAGA |
| 66R | atCCGCTCGAGTATGCACGATTGAGATAGTAAAATGCT |
| 67F | CCGGAATTCATGACCTTTCCAGTGTCCCA |
| 67R | atCCGCTCGAGGGCATCTGTTCTTGGACGTTT |
| 68F | CCGGAATTCATGTCCAGAGACAGGGAGAG |
| 68R | CGCGGATCCATCAAAATATGGGACATTATTTTCACTTGA |
| 69F | CCGGAATTCATGGAGTTTTTAGACGAGGACG |
| 69R | CGCGGATCCACTTTCAGAACCAGCTTTCGAG |
| 70F’CZ | CCATGGAGGCCAGTGAATTCGCCAAACCAGAGATTCATAAAACCA |
| 70R”CZ | CGATTCATCTGCAGCTCGAGATTCATGCTCCTGGGCTGGAC |
| 71F | CCGGAATTCATGTTAAGGTCAAAAAACCAGTGTTTATAC |
| 71R | CGCGGATCCAGAGATCCCCACACCAAGAAAGT |
| 72F | CCGGAATTCATGGCTGTTGATCTAGTTGGGTACT |
| 72R | CGCGGATCCTGTTGACTGGAACACGTGTC |
| 73F | CCGGAATTCATGGCAGGAAATCCTCCACCT |
| 73R | atgCGAGCTCTGAAGGGGTGTTCGATTCGG |
| 74F | CCGGAATTCATGGATGATGATGCGTTGAA |
| 74R | CGCGGATCCCCTGATGGTGACAAACTTGTTG |
| 75 | CDS sequence was synthesized by GENERAY company |
| 76 | CDS sequence was synthesized by GENERAY company |
| 77F | CGCGGATCCatATGGGACGACATTCTTGTTG |
| 77R | atCCGCTCGAGTATCTGGTGGAAGACATCAA |
| 78F | CCGGAATTCATGACATGGTGCAATAATAACTCAGAAG |
| 78R | CGCGGATCCCTGCTTTCTCTGAAGCTTTCC |
| 79F | CGCGGATCCatATGGGGAGGCAATCTTGCGGTTA |
| 79R | atCCGCTCGAGCGAGTGTAGTGTCTGTTGATTGTGTTC |
| 80F | GGGAATTCCATATGATGGAAAAAGACAAGTTGTTTATGAGCGAGGG |
| 80R | atgCCATCGATTAGCTCAATTTTCATCTGAGTTGGTGGCA |
| 81F | CCGGAATTCATGATTCACAAACGTGCTTTTTCTG |
| 81R | CGCGGATCCCTCTTGACTGGAGTGTCTCCCATCA |
| 82F | CCGGAATTCATGGGTGCACCGAAACAGAAAT |
| 82R | CGCGGATCCAAACAAATCATTCAGAAAAGATGCAGTG |
| 83F | CCGGAATTCATGGCAACTACCTCCCAAGCTA |
| 83R | atCCGCTCGAGAAAGAAGAAGTTATTAAACTGAGATGGAG |
| 84F | CCGGAATTCATGATGGGTTTTGGAACTACTGATGATC |
| 84R | CGCGGATCCGCAAGCTGCGGATGAGTGGGTAT |
| 85F | CCGGAATTCATGGATGAGAATATGTCCAATTTAACTTCAGC |
| 85R | CGCGGATCCACCTTGCCAAGGTGGTGGTAGATTT |
| 86F | TCCCCCGGGtATGGGTTCTGCTGAAGAGAAAATC |
| 86R | CGCGGATCCTTCATGGCATTGGATTGGTCTA |
| 87F | CCGGAATTCATGGTTTGTACCACTAATGATTTATCAGC |
| 87R | CGCGGATCCGAAGGAATTAGAGCAAAGGGGAGGAA |
| 88F | CCGGAATTCATGAAAGATTATAGAATGGATGATGGGGA |
| 88R | atCCGCTCGAGATGAGCTTGAGAGGCAGATGGC |
| 89F | TCCCCCGGGtATGTACTTGTCAGAGAAGCCTCG |
| 89R | CGCGGATCCACTCACGCCTCGGTATATAGCAT |
| 90F | CGCGGATCCatATGGATTTGGACTATAACGGAGG |
| 90R | atCCGCTCGAGAGTTGTGGTAGTTGGTACAGCGAC |
| 91F | CCGGAATTCATGGCTATGAAAACTTTTTATCTCAAAG |
| 91R | CGCGGATCCTTTGAGGTTGCTGCAGTTGTCA |
| 92F | TCCCCCGGGtATGCTGCAATCAGGGTATGATATC |
| 92R' | CGCGGATCCACTGTCATGGATGAAGTTCCACT |
| 93F | CCGGAATTCTGGATGTCGAAGAAGAAGAAGAAG |
| 93R | CGCGGATCCTCTACGGCGGGAAAAGTTTTG |
| 94F | TCCCCCGGGtATGGGCGGGTGGAAAGATCAA |
| 94R | CGCGGATCCTGGGATGCGAAAAGGGGACT |
| 95F | TCCCCCGGGtATGGCTGGGATTGATGATAACGTT |
| 95R' | atCCGCTCGAGTGCAGGAGGCCTCTCAAATCTA |
| 96F | CCGGAATTCATGGGTAGCAGTGATATGGATAAAACA |
| 96R | CGCGGATCCTGTTTGTCCAGTCTGTTGTGTATCA |
| 97F-CZ’’ | TACCAGATTACGCTCATATGAATGGGTCTTCTTTAGATTGTCC |
| 97R-CZ’’ | CGATTCATCTGCAGCTCGAGCATGACCATAAGCATCACTCAG |
| 98F | CCGGAATTCATGTCCTCCGCCCTTGGCTA |
| 98R' | CGCGGATCCTTTGGCGCACTTGTCAGGTG |
| 99F | CCGGAATTCATGGATGGTTCTCCATCTAGAA |
| 99R | CGCGGATCCGTGAAAGACGAAGATGTGGGGTA |
| 100F | CCGGAATTCATGGAAACTCGAGATCCACTATCTA |
| 100R | CGCGGATCCAAATGTGGTGCTACTTTCACTTGCA |
| 101F”CZ | CCATGGAGGCCAGTGAATTCGCGAAGAAGAAGACACCAGGAGA |
| 101R”CZ | CGATTCATCTGCAGCTCGAGCGTTTTGATTTGGCCCCAAC |
| 102F | CCGGAATTCATGGAGTGGAATGGCAAA |
| 102R | CGCGGATCCGTGAGACGTCAAGGTATGCA |
| 103F”CZ | CCATGGAGGCCAGTGAATTCGATCCAACAAAACCCCACAAC |
| 103R”CZ | AGCTCGAGCTCGATGGATCCCATGTAGTCTGAGAGATTGTGAGAATCTG |
| 104F | CCGGAATTCATGGCAGGAAATCCCCCACCT |
| 104R | CGCGGATCCTGAGGGGGGGTTCGATTCG |
| 105F | CCGGAATTCATGGGACAGAATTTAGCTACTGCT |
| 105R' | atgCGAGCTCTGACACAAGAGGCTGTATTTCTGA |
| 106F | CCGGAATTCATGTCTAAAAGGGTACTTTGCAAGT |
| 106R | CGCGGATCCCCTTATTTGTAAGCTACCGAGGA |
| 107F | TCCCCCGGGtATGGATAGACGTCGCAGGAA |
| 107R | CGCGGATCCAGAGGTATTAGGAATTACATCTCTTTTTTG |
| 108F | CCGGAATTCATGGAGAAGCACAAGTGCAAA |
| 108R | CGCGGATCCGTCCCACCCTCTCTGGAATA |
| 109F | CCGGAATTCATGGAACCTAAGAGCCCAAAT |
| 109R | CGCGGATCCCTGCCTTGATCCTTGGGAAT |
| 110F | CCGGAATTCATGATGGAAAACAAAAGAAGCCCT |
| 110R | CGCGGATCCATGAAATGGTGAAACGTCCTTATTG |
| 111F-CZ | CCATGGAGGCCAGTGAATTCATGAAGCCATCAAATAACATATGGATTC |
| 111R-CZ | CGATTCATCTGCAGCTCGAGCGTTTCACTGACATTGTTAGGGG |
| 112F | CCGGAATTCATGAATAGTGTCTTCTCAGTGGACGA |
| 112R | atCCGCTCGAGTTTCTTTTTGTAATCCTTCGCAACC |
| 113F | CCGGAATTCATGATTTTATCTGTGAGAGAGTTAAAGCAG |
| 113R | atCCGCTCGAGGCCAGCAGCCACAGCATCA |
| 114F | TCCCCCGGGtATGCAGAAAGGTGGAAAGAGTAAATGTGCA |
| 114R | CGCGGATCCTGGCCCATGGCCAGTGCCA |
| 115F | CCGGAATTCATGTGCACTAGAGGACATTGGA |
| 115R | atCCGCTCGAGTGATGAGGATTTGCCATTAACAG |
| 116F | TCCCCCGGGtATGGTGTTGCAGAAGAGATTAGATTACGGA |
| 116R | atCCGCTCGAGGAGGAGAAGGCAAGTCTCTGCAT |
| 117F | CCGGAATTCATGGGAAGGCAACCATGTTGT |
| 117R | CGCGGATCCCAAGAGCCCATTTGTCCAAGA |
| 118F | GGAATTCCATATGATGGGTGCTCCCAAGCAGAAGT |
| 118R | CCGGAATTCTGTAGACAGGAAGATTGTTATGATACAG |
| 119F | CCGGAATTCATGTCCAGAGAAAGGAAGAGATTTGA |
| 119R | CGCGGATCCACCAAAAAAGGAGACATTTTCAGTC |
| 120F | CCGGAATTCATGGAAACAACGCCTTGTGCGA |
| 120R | TCCCCCGGGGGTAAAACAAGCTTCGCCTTCT |
| 121 | CDS sequence was synthesized by GENERAY company |
| 122F | CCGGAATTCATGAATACCTTTTCGCATGTTCC |
| 122R | CGCGGATCCCTTCCACAGATCAATTTGACAACTG |
| 123F | GGAATTCCATATGATGTGGTCATCGCCAGGA |
| 123R | CGCGGATCCAAATGGAGCTGTTGAGGTTCTA |
| 124F | CCGGAATTCATGGCTAAACAACAGCCGCT |
| 124R | CGCGGATCCCCTCCTTGATCTCTTCTTCGCT |
| 125F | TCCCCCGGGtATGAAAGAGACAGAGAAGAAGAGCT |
| 125R | atCCGCTCGAGGCATTTCGCATCCCTGTGA |
| 126F | TCCCCCGGGtATGGAGACCTCCTCCTCCTCCT |
| 126R' | atCCGCTCGAGCATCAAACATACCATTGACCTCCCT |
| 127F | CCGGAATTCATGGCAGCCGCTCCCCAA |
| 127R | atCCGCTCGAGAAAGAAGAAATTACTAAGCTGAGAAGGAG |
| 128F | TCCCCCGGGtATGGAGGAATATTCTTTGGCCGGT |
| 128R | CGCGGATCCAGAAGATATACCCACACCAAGAAAATCT |
| 129F | CCGGAATTCATGGAGGGAGTTGTAGTGAAAGAA |
| 129R | CGCGGATCCAATCCAGACACCCCTCCGA |
| 130F’ | CCGGAATTCATGGACAACATTAACTCTCGCTCTGAGGTTC |
| 130R’ | CGCGGATCCCACGTTCTGCGGATATGGATACGAAGAA |
| 131F | CCGGAATTCATGGAGAGGCATAAATGCAA |
| 131R | atCCGCTCGAGTGCATCAGAGACCACACTAAATTCA |
| 132F | CCGGAATTCATGACAGAAAACATGAGTATATCTGTGA |
| 132R | CGCGGATCCTACACTAGTGTTTGGCAAGTGC |
| 133F | GGAATTCCATATGATGTCCACCGACGATTTCC |
| 133R | CGCGGATCCACTAATCCTTCCTGCTTGATCAA |
| 134F | CCGGAATTCATGTCTTCCTCCTCCTCCTCT |
| 134R' | CGCGGATCCAAATTGTAGGTTTGCCGAGCA |
| 135F’CZ | GCCAGTGAATTCCACCCGGGtCTGGAAGTGTATAACCAAGGCATG |
| 135R’CZ | AGCTCGAGCTCGATGGATCCTGCAATCCTTGGAATTCTGTTCTC |
| 136F | TCCCCCGGGtATGGAGGAATCTTTGGCTGGTA |
| 136R | CGCGGATCCAGAAGATATACCCACACCAAGAAAATCT |
| 137F | CCGGAATTCATGTCTAAAAGGTTACTATGCAAGTTC |
| 137R | CGCGGATCCGCTTCCCGAAATCGAATCT |
| 138F | GGAATTCCATATGATGGAAGAATCAAGTGAAGTGCA |
| 138R | CCGGAATTCTCTTTCTGACAGGTGATCACACA |
| 139F | CCGGAATTCATGGCTAGCCACAGAATTGGAGA |
| 139R | CGCGGATCCAAAGTTGGAGAAATGATTTTGTGAAGGT |
| 140F | TCCCCCGGGtATGGCAGGGAGTGAGAGCTT |
| 140R | CGCGGATCCGCAAGGTTTGATTTCAAACCCA |
| 141F | CCGGAATTCATGGATTTTGCTGAGAAAATACAAAGGT |
| 141R | CGCGGATCCCGTTGTTGTAGTATGTGTGGAGGAA |
| 142F | CCGGAATTCATGGATGAACTAAAGATAGAAGAGCGCT |
| 142R | CGCGGATCCGGTAACCCCCATCTTTGTTGCA |
| 143F | TCCCCCGGGtATGGTCTCGTCAGAGGAAGTTTCT |
| 143R | CGCGGATCCTGGGAAATGGGAGATGGCAT |
| 144F | CCGGAATTCATGTCTAGCAGAGGCCATTGGA |
| 144R | atgCGAGCTCCAATCCACTAACTTGGTTTCCA |
| 145F | TCCCCCGGGtATGAAGGTTCCTTCAAATGGGT |
| 145R' | atCCGCTCGAGTTGTAACAACAGGAGTGGGGACT |
| 146F | TCCCCCGGGtATGGACCCTCTTGTCATAAAATTGA |
| 146R' | atCCGCTCGAGTGGTAGTAGGTGCTTTACAAGCCA |
| 147F | CCGGAATTCATGGGTGTTCAAACAATGGCAT |
| 147R | CGCGGATCCGAAAGAAGTAGAACTTGTTCTTCTAAGCTGA |
| 148F | CCGGAATTCATGACAGAAAACATGAGTATATCTGTTAATG |
| 148R | CGCGGATCCTGCACCTGTGTTTGACAACTG |
| 149F | CCGGAATTCATGTCAACAGCACCATCTTCTGAT |
| 149R | atCCGCTCGAGGCTGACACAAGCTTCGCTTTC |
| 150 | CDS sequence was synthesized by GENERAY company |
| 151F | CCGGAATTCATGAAGAGAGGTCTGCACGAGAGA |
| 151R | atCCGCTCGAGAAAGAAACAATTGACCATAGGAGCTGTC |
| 152F | TCCCCCGGGtATGAATACTTTTACACATGTTCCTCCTG |
| 152R | CGCGGATCCTTTCCATAGATCAATTTGACAACTGGA |
| 153F | TCCCCCGGGtcGGACGCAGAATGCTGTGAGT |
| 153R | atgCGAGCTCTTCCAGGCACTGGATTTCTCA |
| 154F | CCGGAATTCATGGAAGCACCGGAATTGTATG |
| 154R | CGCGGATCCGATCATCCGCCTAAAATCGGGT |
| 155F | TCCCCCGGGtATGCCAGGCCTTCAAAGGCCA |
| 155R | atCCGCTCGAGTTGGTGAGCAAAATTGAGAGTTTCCTC |
| 156F | CCGGAATTCATGGAAGACATAGAAGAGGAGGA |
| 156R | CGCGGATCCTACACAATGCCATGCAGAGG |
| 157 | CDS sequence was synthesized by GENERAY company |
| 158F | TCCCCCGGGtATGGGACGACATTCTTGTTGT |
| 158R | CGCGGATCCTATCTGGTGGAAGCCATCGA |
| 159 | CDS sequence was synthesized by GENERAY company |
| 160F | TCCCCCGGGtATGGCCACCGACGATTTC |
| 160R | CGCGGATCCACTAATCCTTCCTGCTTGATCAATC |
| 161F | CCGGAATTCGAATCAGGGGCATTTCCTG |
| 161R | CGCGGATCCATATGAACCCCATATATCATCTACGTC |
| 162F | TCCCCCGGGtATGAGGAAGCCGGATCTAATG |
| 162R | CGCGGATCCTAAAACTTGGAAATCAAGGAAAGGA |
| 163F’CZ | TACCAGATTACGCTCATATGGCCTCTGGGAAGAACAACGATATAA |
| 163R’CZ | CGTATCGATGCCCACCCGGGACTATATGGGTGCTTGTCAGTGCTAT |
| 164F | CGCGGATCCatATGAAGAAGAATCAAGAAAAGGTGCA |
| 164R | atCCGCTCGAGGTTGGCTATAAGACCCACCAGT |
| 165F’-CZ | CCATGGAGGCCAGTGAATTCTCTGATAAGTTAGGACCTCAATGGAAG |
| 165R’-CZ | AGCTCGAGCTCGATGGATCCCTGCGAACCATAGGAGATGATACTAC |
| 166F-CZ | GCCAGTGAATTCCACCCGGGtATGTGTCAATGGCCTAATCAGTCA |
| 166R-CZ | TCGATGGATCCCGTATCGATAGCAACGTTCTGATCAAGCTG |
| 167F | CCGGAATTCATGGATTTTGCAGAGAAAATGCAAC |
| 167R | CGCGGATCCAAACACTGGGGATGTCGTGGTA |
| 168F | CCGGAATTCATGGACCCCAGCCCCTCC |
| 168R | CGCGGATCCGACCTGTAGCACAACTGAACTGGAAG |
| 169 | CDS sequence was synthesized by GENERAY company |
| 170F | CCGGAATTCATGCCGCAACTGACGGAG |
| 170R | atCCGCTCGAGAGTAGGACATGCCTCAACAGGTGA |
| 171 | CDS sequence was synthesized by GENERAY company |
| 172F | CCGGAATTCATGGGTCGCTCACCTTGTTGC |
| 172R | CGCGGATCCCTTCCAACAATAATATCTATGATTCTGGC |
| 173F | TCCCCCGGGtATGGGTGATTCTGGAAAGACGT |
| 173R' | atCCGCTCGAGACTTCTTCAAGCTCACATCATCACT |
| 174F | TCCCCCGGGtATGGACAGCACTTCTAAGCAAGC |
| 174R | CGCGGATCCGACATATTTGCAATCCGAATCCTC |
| 175F | CCGGAATTCATGAGTACTACAACAACAAACCCTATAAATAGC |
| 175R | CGCGGATCCTTTAGAGATAGATGATGATGCTAATGCGT |
| 176F | GGAATTCCATATGATGGACTTCCATCTGAAGCAAT |
| 176R | CCGGAATTCGAAACAGTGGGGCGACATAA |
| 177F | CCGGAATTCATGTCTTCTTCCAACTCTCCATGT |
| 177R | CGCGGATCCACAAGAAGGACCAACACTCCTCCT |
| 178F | TCCCCCGGGtATGTCGAGCTCACAAGCACCA |
| 178R | CGCGGATCCCCCGGCAGTAGTGTTAGCAG |
| 179F | GGAATTCCATATGATGCAGAAAGGTAAGAAGAGTAAATG |
| 179R | CCGGAATTCGTTTTCTCTATCAGCCTTTGTCTTTC |
| 180F | CCGGAATTCATGGTGATGATGGAAGAAGAAG |
| 180R | CGCGGATCCGGTAGGTAGGGCATGGCTC |
| 181F | CCGGAATTCATGGGCAGATCTCCTTGTTGT |
| 181R | CGCGGATCCAGCATCCAAAGGTCTGTAAAATCT |
| 182F | CCGGAATTCATGGAAAGAACTGATTCCTCCA |
| 182R | CGCGGATCCAGAGTACCAATTCATGCTTGGA |
| 183F | CCGGAATTCATGACCATTGAAGGATCAGAGCCA |
| 183R | CGCGGATCCACTGTCACTTCCTTTCATATCCAATTTC |
| 184F | TCCCCCGGGtATGGGGAGGTCTCCCTGTT |
| 184R | CGCGGATCCGGAAATATCATGCATAATAGGACCTTC |
| 185F | CCGGAATTCATGGCAGATGAGTACACAAGCA |
| 185R | CGCGGATCCTCTGTAAGTCCCTCCAAATGTAGG |
| 186F | CCGGAATTCATGAGTACCGCAACTACAAACCCT |
| 186R | CGCGGATCCTTTAGAGATAGATGGTGATGCTAATGTG |
| 187F | TCCCCCGGGtATGGGTGATTCTGGAAAGAAATCA |
| 187R' | atCCGCTCGAGTACTTATTGACCTCGGCTTCTTCCT |
| 188F | TCCCCCGGGtATGAAGGAAGCTTCAGGTGTCT |
| 188R | atCCGCTCGAGAATATGAAGCATGATGTCGGGA |
| 189F | CCGGAATTCATGCAAGATTCTCAACCAACCA |
| 189R | CGCGGATCCAGGGAATTTCCAAAGGTTCTGGT |
| 190 | CDS sequence was synthesized by GENERAY company |
| 191F | CCGGAATTCATGGGGGAAGAAGTGAAAATGAGT |
| 191R | atCCGCTCGAGCATGTTGTCATTAGGTGCAACTTGA |
| 192F | CCGGAATTCATGAAGAAGAATCAAGAAAAGATGC |
| 192R | CGCGGATCCGTCTAGATCAACAATAATGTTTTTGTTCATCAG |
| 193F | CCGGAATTCATGGGAAGGCAACCGTGTT |
| 193R | CGCGGATCCCATGAGCCCATATGTCCAATAATCT |
| 194F | CCGGAATTCATGGCATTCACTGGAACCCT |
| 194R | CGCGGATCCAGATTGTTCTTCTGACTTATCCTCAGCA |
| 195F | CCGGAATTCATGAGCAGCATCTCTACTTCTGA |
| 195R | CGCGGATCCGCCTAGCAAATCTGAAGAACTTG |
| 196 | CDS sequence was synthesized by GENERAY company |
| 197 | CDS sequence was synthesized by GENERAY company |
| 198F | CCGGAATTCATGAATACCTTCTCGCATGTCC |
| 198R | CGCGGATCCCTTCCATAGATCAATTTGGCAAC |
| 199F | TCCCCCGGGtATGTGTATAGCCATGGACAGGAACT |
| 199R | CGCGGATCCAAAACAGCCAAGTCGAAGCTC |
| 200F | CCGGAATTCATGGGAAGGGGTAGGGTTCA |
| 200R | CGCGGATCCTGCTCCGTAACCTCCAAGGT |
| 201F | CCGGAATTCATGGAGAATGGTTGGAGCTG |
| 201R | CGCGGATCCTGAGAAAAATCCTGGTGAGTCA |
| 202F | CCGGAATTCATGGGTAAAGGGAGAGCG |
| 202R | CGCGGATCCTAAAGAAGGCCACAAATGAAA |
| 203F | CCGGAATTCATGAGGAAGCCGGATCTAGTG |
| 203R | CGCGGATCCTTCTACGTGGAAATCAAGTAAAGGA |
| 204F | CCGGAATTCACAACTGCTACACGAGCCACT |
| 204R | CGCGGATCCAAGCCAGAGATCATTATTAGGATCTTC |
| 205F | CCGGAATTCATGACAAGCTCAACAGTACCTTTTCCA |
| 205R | CGCGGATCCATTATAATATCCTCCATCAACTCCGAGGA |
| 206F | CCGGAATTCAGGACGATGGTATCGCAAACTT |
| 206R | CGCGGATCCAGAAGACGAGCTCGAGGAATC |
| 207F | GGAATTCCATATGATGGTTAGGTCTCCTCGTAGCCA |
| 207R | TCCCCCGGGTTCCAGAATATCTTTCCAGTAGGAGCT |
| 208F | TCCCCCGGGtATGTCGTCTTCGAACTCTCCA |
| 208R | CGCGGATCCACAAGATGGACCAACAGTACTTCT |
| 209F | CCGGAATTCATGGAAGCCAAGAAAACAAGAGGA |
| 209R | CGCGGATCCCACGCCCCAATTACTTCCATGA |
| 210F | CCGGAATTCATGGAAGGAGAGTGTTATACATCACCA |
| 210R | CGCGGATCCCTCTTCATCACAATTCTCTGGGTACT |
| 211F | TCCCCCGGGtATGGAAATTCATTTCCAGCAAC |
| 211R | atCCGCTCGAGTAGTGGTGGAAGATCCCATAAAGCT |
| 212F | CCGGAATTCATGGAAGGCATGGAAGATCAA |
| 212R | CGCGGATCCTGCACAAATACGCCATGCAGA |
| 213F’CZ | GCCAGTGAATTCCACCCGGGTCTGGAGTAAAAGATCAATGGGAGA |
| 213R'CZ | AGCTCGAGCTCGATGGATCCTGTTTTGAACAATGTACGCGTGTAC |
| 214F | CCGGAATTCATGGTCAAGAAATTTCCACAGGGA |
| 214R | CGCGGATCCTACTAAAAGGCCAAGTCGAAGCTC |
| 215F | CCGGAATTCATGGTGAACAAAAAGCCAAGCA |
| 215R | CGCGGATCCAAAAATCCCATGCCCATAACCA |
| 216F | CCGGAATTCATGGAACCTCAACAACAACAACAC |
| 216R | CGCGGATCCATAATCTTGTTGCTGGCCATTGT |
| 217F | CCGGAATTCATGGACTTGACCTCTAGCCCCCA |
| 217R | CGCGGATCCAGATGAAGTAGAAGACCCATGAGGAGT |
| 218F | GGAATTCCATATGATGGTCAAATCGGCCTCTATTGAT |
| 218R | CCGGAATTCATTACTGCCTTCCAGTTCCTGCA |
| 219 | CDS sequence was synthesized by GENERAY company |
| 220F’CZ | CCATGGAGGCCAGTGAATTCCTCTTCTTCAAAGAGTACAATTCCTGGA |
| 220R’CZ | AGCTCGAGCTCGATGGATCCTGGATGAGCAATGACTGGTTTTCT |
| 221 | CDS sequence was synthesized by GENERAY company |
| 222F | GGAATTCCATATGATGCTTCAGCCATATCTTCACGA |
| 222R | CCGGAATTCTTTGTTCACGTTGCGATCCCT |
| 223F | CCGGAATTCATGTGCAGTCCTGAACTCCTTC |
| 223R | CGCGGATCCAGAGCCCTGAAAGCTCTGCTG |
| 224F | CCGGAATTCATGGCCACAACTTCATCTAGC |
| 224R | CGCGGATCCAGAATAGCTCCACAGACTTTCTCC |
| 225F | CCGGAATTCATGAGTGCATCAAAAAACACAAACC |
| 225R | CGCGGATCCTGGATGTAAGGGTGCTAGTCG |
| 226F | TCCCCCGGGtATGGCTATACAAAGTGATCATCTTGC |
| 226R | CGCGGATCCGAAAGAGGAAAACATACTGTCCAG |
| 227F’ | CCGGAATTCAAGTACGAAGAGCCTGAAACACTGA |
| 227R | CGCGGATCCTGCAAAGTTGGTTCTTGCAT |
| **Primers for amplification of monolignol pathway gene promoters from *P. trichocarpa* for Y1H (Restriction enzyme sites are underlined)** | |
|  |  |
| pCAld5H2-F | CGGGGTACCACCGAGAATGTGTATGAGGTTGAAG |
| pCAld5H2-R | AtCCGCTCGAGCGCAACTTTCCGATGCTGCTAAG |
| pCAld5H1-F | CGGGGTACCCACCCTCTTGCAATTAGTGCTTC |
| pCAld5H1-R | ATCCGCTCGAGCAACTTTCAGATGTTGCTAAGCTGAG |
| pCOMT2-F | CGGGGTACCCGAAGCAGCAGAAGAGAATGGT |
| pCOMT2-R | AtCCGCTCGAGGTGGTTGGTCCCTACTCTCTAC |
| pCAD1-F | CGGGGTACCGGTTAACGGAAAACACTTTCCAGT |
| pCAD1-R | AtCCGCTCGAGTTTCTTGAAACAATGAGGCTAAGAG |
| pC3H3-F | CGGGGTACCCGGCCACCGTGGTCCTTATCTC |
| pC3H3-R | TACGCGTCGACCTAATTTTTCTGAAGGGCCTTGGCAG |
| pCCR2-F | CGGGGTACCACCCTTTCTCCGAGCAACTTAAAG |
| pCCR2-R | ATCCGCTCGAGGACAAAGAAGAGAGGAGGCGTG |
| pPAL4-F | CGGGGTACCCCAATTATTTGACTGGCTCGTGTG |
| pPAL4-R | TACGCGTCGACTGCGAGGCTCTAAATGTATGTAGG |
| pHCT1-F | CGGGGTACCCACCCTCTATAAATGCCCGTGATC |
| pHCT1-R | ATCCGCTCGAGGGTGAGATTTCATCGCGCAAGC |
| pPAL2-F | CGGGGTACCCCATACCATACAATGACTTGCTCATC |
| pPAL2-R | ATCCGCTCGAGCTTCAGGTCACCAAAAATCCCTG |
| pC4H2-F | CGGGGTACCTGAGGCTGATATTGGTACGATGTGC |
| pC4H2-R | ATCCGCTCGAGAGGAGAGACTAGGTGGTTGGTG |
| p4CL5-F | CGGGGTACCGCTCCTGCAAGTTTAATTCTCTCTCC |
| p4CL5-R | ATCCGCTCGAGGTTGCAGAGGGAGAGGGTGGA |
| **Primers for the amplification of seven fragments of *P. trichocarpa CAld5H2* promoter for Y1H (Restriction enzyme sites are underlined)** | |
|  |  |
| C5-1F | CGGGGTACCACCGAGAATGTGTATGAGGTTGAAG |
| C5-1R | AtCCGCTCGAGAGCAAGAGAAAATTGTTGTAAGTTAAATAG |
| C5-2F | CGGGGTACCTACAACAATTTTCTCTTGCTGACTATAG |
| C5-2R | AtCCGCTCGAGAAACCGATTACTAGCATCCAACA |
| C5-3F | CGGGGTACCGTTGGATGCTAGTAATCGGT |
| C5-3R | AtCCGCTCGAGATATAAAAATAAAATATTTATTACAAATTTAAAC |
| C5-4F | CGGGGTACCGAACTTATAAATGTTTAAATTTGTAATAAA |
| C5-4R | AtCCGCTCGAGTAATATATTACCAACAAAAAAACTGTA |
| C5-5F | CGGGGTACCTTTTTGTTGGTAATATATTATATTCATCGA |
| C5-5R | AtCCGCTCGAGCGAAAACAAGACAGAACAATTTCA |
| C5-6F | CGGGGTACCATTGTTCTGTCTTGTTTTCGTTTCATC |
| C5-6R | AtCCGCTCGAGAAATAAGTTTATATTTTCTCTTGTGAAAGGT |
| C5-7F | CGGGGTACCGAGAAAATATAAACTTATTTGATTTCAGCA |
| C5-7R | AtCCGCTCGAGGGATGGTTTATGTGTTGGTGC |
| **Primers for *CAld5H1/2*, *BLH6a/b*, *BZIP34,* *bHLH59* *in situ* hybridization in *P. abla* × *P. glandulosa* (Restriction enzyme sites are underlined)** | |
|  |  |
| CAld5Hyw-S-F | GAATTCTAATACGACTCACTATAGGGGGATGTGATGTTTGGTGGGAC |
| CAld5Hyw-S-R | ACCAGCAACCTCAGCATCTTC |
| CAld5Hyw-AS-F | GGATGTGATGTTTGGTGGGAC |
| CAld5Hyw-AS-R | GAATTCTAATACGACTCACTATAGGGACCAGCAACCTCAGCATCTTC |
| BLH6yw-S-F | GAATTCTAATACGACTCACTATAGGGAAGGTGATATGGGAACCTCTGAAG |
| BLH6yw-S-R | TCGGAGTGAGCCATAGCATCAG |
| BLH6yw-AS-F | AAGGTGATATGGGAACCTCTGAAG |
| BLH6yw-AS-R | GAATTCTAATACGACTCACTATAGGGTCGGAGTGAGCCATAGCATCAG |
| BZIP34yw-S-F | GAATTCTAATACGACTCACTATAGGGAGAATGGATATCTGTGAGAGTTGAAGC |
| BZIP34yw-S-R | TGACTCCAGTCCACTACAAGTTTC |
| BZIP34yw-AS-F | AGAATGGATATCTGTGAGAGTTGAAGC |
| BZIP34yw-AS-R | GAATTCTAATACGACTCACTATAGGGTGACTCCAGTCCACTACAAGTTTC |
| BHLH59yw- S-F | GAATTCTAATACGACTCACTATAGGGACCAACCTAATGCTCCGACAC |
| BHLH59yw- S-R | GATGTAGAAATGGGAAACCAGACC |
| BHLH59yw- AS-F | ACCAACCTAATGCTCCGACAC |
| BHLH59yw- AS-R | GAATTCTAATACGACTCACTATAGGGGATGTAGAAATGGGAAACCAGACC |
| **Primers for transcriptional activation/inhibition assays in yeast** | |
| BLH6a-BD-F | TCAGAGGAGGACCTGCATATGATGGCTACCTATTATACTAGTTCAAATAATCAAAG |
| BLH6a-BD-R | TCGACGGATCCCCGGGAATTCAGCTACGAAATCATGAAATACGTGG |
| BLH6a-BD-VP16-F | TCAGAGGAGGACCTGCATATGATGGCTACCTATTATACTAGTTCAAATAATCAAAG |
| BLH6a-BD-VP16-R | ATCGGTCGGGGGGGCGAATTCAGCTACGAAATCATGAAATACGTGG |
| **Primers for effector-reporter-based repression assays in tobacco leaves** | |
|  |  |
| pCAld5H2-To-F | CGGTATCGATAAGCTTAGCGAAGATGTGTGTGAAGTTGAAG |
| pCAld5H2-To-R | TGTAAAAATAAAGCTTCGCAACTTTCTGATGTTGCTAAG |
| BLH6agw-F | caccATGGCTACCTATTACACTAGTTCGAATAATC |
| BLH6agw-R | AGCAACGAAATCATGAAATACATGG |
| BLH6bgw-F | caccATGGCTACCTATTATACTAGTTCAAATAATCAAAG |
| BLH6bgw-R | AGCTACGAAATCATGAAATACGTGG |
| BLH2gw-F | caccATGGGCATAGCTACACCTCCA |
| BLH2gw-R | ACAGCCCCCAAAATCTCTAAC |
| **Primers for transgenic production** | |
| SRDX-F | GATCCttggatcttgatttggaacttagacttGGTTTCGCTTGAGAGCT |
| SRDX-R | CTCAAGCGAAACCAAGTCTAAGTTCCAAATCAAGATCCAAG |
| 121BLH6a-F | CGGGGGACTCTAGAGGATCCCTGGGCGGTATGTGATTGAA |
| 121BLH6a-R | AAATCAAGATCCAAGGATCCAGCTACGAAATCATGAAATACGTGG |
| **Primers for monolignol pathway gene expression by qRT-PCR in *P. abla* × *P. glandulosa transgenics*** | |
|  |  |
| MUT18-rF | GCCAGGGTTTATCTCTCAGTC |
| MUT18-rR | GGACTCAAGAACGAAGTAAGGC |
| CAld5H2-rF | GCACCAACACATAAACC |
| CAld5H2-rR | CGAGAGAGGAGACCTAAG |
| CAld5H1-rF | AATTAATCAGTGGGGGTTG |
| CAld5H1-rR | TGCAAAGATTGGAGAAGAG |
| 4CL3-rF | GGAGATGTCTACACCTATGCTGA |
| 4CL3-rR | CTTGGTAGGAAGAGCATGATCAC |
| 4CL5-rF | AGAGCCAAGTTTCCTCAGGC |
| 4CL5-rR | ATTCCTAACCACAGTCCCGC |
| C3H3-rF | GAAGCGATTCGAGAATGCCG |
| C3H3-rR | CGGAAACATCCAACGAAGCC |
| C4H2-rF | GGCAACTGGCTTCAAGTTGG |
| C4H2-rR | ACAACAAGATTGCGTTGGCC |
| CAD1-rF | TTCTGACAAGAAGCGGGAGG |
| CAD1-rR | GGTGAACCACAGGCACAGTA |
| CCR2-rF | TGCTTCTCCCGTCACAGATG |
| CCR2-rR | GAACACCACTCGTCGGACTT |
| COMT2-rF | CTTTGAAGGCCTCACGTCCT |
| COMT2-rR | TGACATGGGGCAGATCGAAG |
| PAL4-rF | ATCACCCCATGTTTGCCACT |
| PAL4-rR | TCTCCATTGGGTCCAACTGC |
| HCT1-rF | GCCAAAGCAAAGGAAGGTGG |
| HCT1-rR | TTGACCGACCATCTGTAGCG |
| **Primers for yeast two hybridization assays** | |
| BLH6a-BD-F | TCAGAGGAGGACCTGCATATGATGGCTACCTATTATACTAGTTCAAATAATCAAAG |
| BLH6a-BD-R | TCGACGGATCCCCGGGAATTCAGCTACGAAATCATGAAATACGTGG |
| BLH6b-AD-F | TCCCCCGGGTATGGCTACCTATTACACTAGTTCGAATAATC |
| BLH6b-AD-R | ATCCGCTCGAG AGCAACGAAATCATGAAATACATGG |
| BLH2-AD-F | TCCCCCGGGTATGGGCATAGCTACACCTCCA |
| BLH2-AD-R | ATCCGCTCGAG ACAGCCCCCAAAATCTCTAAC |
| **Primers for LCI assays of 12 TFs (*P. abla* × *P. glandulosa*)** | |
| BLH6a-cLUC-F | ACGCGTCCCGGGGCGGTACC ATGGCTACCTATTATACTAGTTC |
| BLH6a-cLUC-R | GTTGCTGCAGGTCGAC TCAAGCTACGAAATCATGAAATACGTGG |
| BLH6b-cLUC-F | ACGCGTCCCGGGGCGGTACC ATGGCTACCTATTACACTAGTTC |
| BLH6b-cLUC-R | GTTGCTGCAGGTCGAC TCAAGCAACGAAATCATGAAATACATGG |
| BLH2-cLUC-F | ACGCGTCCCGGGGCGGTACC ATGGGCATAGCTACACCTCCA |
| BLH2-cLUC-R | GTTGCTGCAGGTCGAC TTAACAGCCCCCAAAATCTCTAAC |
| BZIP34-cLUC-F | ACGCGTCCCGGGGCGGTACC ATGTCAAGGCAATCCCTACTTCC |
| BZIP34-cLUC-R | GTTGCTGCAGGTCGAC TCAGTTAAGATTAAGTTTTGCCATCTGCC |
| bHLH59-cLUC-F | ACGCGTCCCGGGGCGGTACC ATGGCAGGAAATCCCCCACC |
| bHLH159-cLUC-R | GTTGCTGCAGGTCGAC TTATGAGTGGGGGTTCGATTCGG |
| MYB69-cLUC-F | ACGCGTCCCGGGGCGGTACC ATGTTAAGGTCAAAAAACCAGTG |
| MYB69-cLUC-R | GTTGCTGCAGGTCGAC TCAAGAGATCCCCACACCAAGAAAG |
| MYB85a-cLUC-F | ACGCGTCCCGGGGCGGTACC ATGGGCAGACAACCTTGTTG |
| MYB85a-cLUC-R | GTTGCTGCAGGTCGAC TTAATGCTTGCCACCCATGTCTAG |
| MYB85b-cLUC-F | ACGCGTCCCGGGGCGGTACC ATGGGTAGACAACCTTGTTGC |
| MYB85b-cLUC-R | GTTGCTGCAGGTCGAC TTAATGCTTGCCAACCATGTCTAG |
| VAL2a-cLUC-F | ACGCGTCCCGGGGCGGTACC ATGGCGTCCTCGTCGTTGTC |
| VAL2a-cLUC-R | GTTGCTGCAGGTCGAC TCAGACAGGGTCATTTTGGCTTTG |
| VAL2b-cLUC-F | ACGCGTCCCGGGGCGGTACC ATGGCGTCGTCGTCGATTAAG |
| VAL2b-cLUC-R | GTTGCTGCAGGTCGAC TTATAGAAGGTCATTTTGGCTTTGGTC |
| NAC75-cLUC-F | ACGCGTCCCGGGGCGGTACC ATGAGTACTAGGTGTAGCATGGCTTC |
| NAC75-cLUC-R | GTTGCTGCAGGTCGAC CTA CCCATGATGATCCTGGTTGC |
| SND2-cLUC-F | ACGCGTCCCGGGGCGGTACC ATGATTTTCTTTTTCTTTTCTTTTTATGTC |
| SND2-cLUC-R | GTTGCTGCAGGTCGAC TCACCATCTTCTCTGAAGCTTTCCTTTG |
| BLH6a-nLUC-F | CACGGGGGACGAGCTCGGTACC ATGGCTACCTATTATACTAGTTC |
| BLH6a-nLUC-R | ACGCGTACGAGATCTGGTCGAC AGCTACGAAATCATGAAATACGTGG |
| BLH6b-nLUC-F | CACGGGGGACGAGCTCGGTACC ATGGCTACCTATTACACTAGTTC |
| BLH6b-nLUC-R | ACGCGTACGAGATCTGGTCGAC AGCAACGAAATCATGAAATACATGG |
| BLH2-nLUC-F | CACGGGGGACGAGCTCGGTACC ATGGGCATAGCTACACCTCCA |
| BLH2-nLUC-R | ACGCGTACGAGATCTGGTCGAC ACAGCCCCCAAAATCTCTAAC |
| BZIP34-nLUC-F | CACGGGGGACGAGCTCGGTACC ATGTCAAGGCAATCCCTACTTCC |
| BZIP34-nLUC-R | ACGCGTACGAGATCTGGTCGAC GTTAAGATTAAGTTTTGCCATCTGCC |
| bHLH59-nLUC-F | CACGGGGGACGAGCTCGGTACC ATGGCAGGAAATCCCCCACC |
| bHLH59-nLUC-R | ACGCGTACGAGATCTGGTCGAC TGAGTGGGGGTTCGATTCGG |
| MYB69-nLUC-F | CACGGGGGACGAGCTCGGTACC ATGTTAAGGTCAAAAAACCAGTG |
| MYB69-nLUC-R | ACGCGTACGAGATCTGGTCGAC AGAGATCCCCACACCAAGAAAG |
| MYB85a-nLUC-F | CACGGGGGACGAGCTCGGTACC ATGGGCAGACAACCTTGTTG |
| MYB85a-nLUC-R | ACGCGTACGAGATCTGGTCGAC ATGCTTGCCACCCATGTCTAG |
| MYB85b-nLUC-F | CACGGGGGACGAGCTCGGTACC ATGGGTAGACAACCTTGTTGC |
| MYB85b-nLUC-R | ACGCGTACGAGATCTGGTCGAC ATGCTTGCCAACCATGTCTAG |
| VAL2a-nLUC-F | CACGGGGGACGAGCTCGGTACC ATGGCGTCCTCGTCGTTGTC |
| VAL2a-nLUC-R | ACGCGTACGAGATCTGGTCGAC GACAGGGTCATTTTGGCTTTG |
| VAL2b-nLUC-F | CACGGGGGACGAGCTCGGTACC ATGGCGTCGTCGTCGATTAAG |
| VAL2b-nLUC-R | ACGCGTACGAGATCTGGTCGAC TAGAAGGTCATTTTGGCTTTGGTC |
| NAC75-nLUC-F | CACGGGGGACGAGCTCGGTACC ATGAGTACTAGGTGTAGCATGGCTTC |
| NAC75-nLUC-R | ACGCGTACGAGATCTGGTCGAC CCCATGATGATCCTGGTTGC |
| SND2-nLUC-F | CACGGGGGACGAGCTCGGTACC ATGATTTTCTTTTTCTTTTCTTTTTATGTC |
| SND2-nLUC-R | ACGCGTACGAGATCTGGTCGAC CCATCTTCTCTGAAGCTTTCCTTTG |
|  |  |
