# Supplementary material for: BEL1-like Homeodomain Protein BLH6a Is a Negative Regulator of CAld5H2 in Sinapyl Alcohol Monolignol Biosynthesis in Poplar
Source: Front Plant Sci. 2021 Jun 25;12:695223. doi: 10.3389/fpls.2021.695223 (PMC8269948; doi:10.3389/fpls.2021.695223)
Supplement: Supplementary Table 2 — 12 TF candidates regulating CAld5H2. [file Table_2.docx]

**Supplementary Table S2** 12 TF candidates regulating *CAld5H2*

| **Gene name** | **JGI ID** | **Arabidopsis ID** | **Annotation** | **Note**  **(Other name)** |
| --- | --- | --- | --- | --- |
| *PagBLH6a* | Potri.004G159300 | [AT4G34610](http://planttfdb.cbi.pku.edu.cn/tf.php?sp=Ath&did=AT2G16400.1) | BEL1-like homeodomain 6 | PtrWBLH3 |
| *PagBLH2* | Potri.005G129500 | [AT4G36870.2](http://planttfdb.cbi.pku.edu.cn/tf.php?sp=Ath&did=AT4G36870.2) | BEL1-like homeodomain 2 | PtrWBLH2 |
| *PagSND2* | Potri.011G058400 | [AT4G28500.1](http://planttfdb.cbi.pku.edu.cn/tf.php?sp=Ath&did=AT4G28500.1) | SND2 | PtrNAC123 |
| *PagBLH6b* | Potri.009G120800 | [AT4G34610.2](http://planttfdb.cbi.pku.edu.cn/tf.php?sp=Ath&did=AT4G34610.2) | BEL1-like homeodomain 6 |  |
| *PagBZIP34* | Potri.008G018400 | [AT2G42380.2](http://planttfdb.cbi.pku.edu.cn/tf.php?sp=Ath&did=AT2G42380.2) | basic leucine zipper 34 |  |
| *PagbHLH59* | Potri.005G053500 | [AT4G02590.3](http://planttfdb.cbi.pku.edu.cn/tf.php?sp=Ath&did=AT4G02590.3) | bHLH family protein |  |
| *PagMYB69* | Potri.005G063200 | [AT4G33450.1](http://planttfdb.cbi.pku.edu.cn/tf.php?sp=Ath&did=AT4G33450.1) | myb domain protein 69 |  |
| *PagMYB85a* | Potri.012G127700 | [AT4G22680.1](http://planttfdb.cbi.pku.edu.cn/tf.php?sp=Ath&did=AT4G22680.1) | myb domain protein 85 |  |
| *PagMYB85b* | Potri.015G129100 | [AT4G22680.1](http://planttfdb.cbi.pku.edu.cn/tf.php?sp=Ath&did=AT4G22680.1) | myb domain protein 85 |  |
| *PagVAL2a* | Potri.006G108300 | [AT4G32010.1](http://planttfdb.cbi.pku.edu.cn/tf.php?sp=Ath&did=AT4G32010.1) | HSI2-like 1 |  |
| *PagVAL2b* | Potri.016G136500 | [AT4G32010.1](http://planttfdb.cbi.pku.edu.cn/tf.php?sp=Ath&did=AT4G32010.1) | HSI2-like 1 |  |
| *PagNAC75* | Potri.018G068700 | [AT4G29230.1](http://planttfdb.cbi.pku.edu.cn/tf.php?sp=Ath&did=AT4G29230.1) | NAC domain protein 75 |  |
