# Supplementary material for: BEL1-like Homeodomain Protein BLH6a Is a Negative Regulator of CAld5H2 in Sinapyl Alcohol Monolignol Biosynthesis in Poplar
Source: Front Plant Sci. 2021 Jun 25;12:695223. doi: 10.3389/fpls.2021.695223 (PMC8269948; doi:10.3389/fpls.2021.695223)
Supplement: Supplementary Table 3 — Summary of interactions among 12 TFs. [file Table_3.docx]

**Supplementary Table S3** Summary of interactions among 12 TFs

|  | BLH6a  -nluc | BLH6b  -nluc | BZIP34  -nluc | bHLH59  -nluc | MYB69  -nluc | MYB85a  -nluc | MYB85b  -nluc | VAL2a  -nluc | VAL2b  -nluc | NAC75  -nluc | SND2  -nluc | BLH2  -nluc |
| --- | --- | --- | --- | --- | --- | --- | --- | --- | --- | --- | --- | --- |
| BLH6a-cluc | √ | √ | √ | √ | X | √ | √ | X | X | √ | √ | √ |
| BLH6b-cluc | √ | √ | √ | √ | √ | √ | √ | X | X | √ | √ | √ |
| BZIP34-cluc | √ | √ | √ | √ | √ | √ | √ | √ | √ | √ | √ | √ |
| bHLH59-cluc | √ | √ | √ | √ | √ | X | √ | √ | √ | √ | √ | √ |
| MYB69-cluc | X | √ | √ | √ | √ | √ | √ | X | √ | √ | √ | √ |
| MYB85a-cluc | √ | √ | √ | X | √ | √ | √ | √ | √ | √ | √ | √ |
| MYB85b-cluc | √ | X | √ | √ | √ | √ | X | X | √ | √ | X | X |
| VAL2a-cluc | X | X | √ | √ | X | √ | X | √ | √ | √ | √ | √ |
| VAL2b-cluc | √ | X | √ | √ | √ | √ | √ | √ | √ | √ | √ | X |
| NAC75-cluc | √ | √ | √ | √ | √ | √ | √ | √ | √ | √ | √ | √ |
| SND2-cluc | √ | √ | √ | √ | √ | √ | √ | √ | √ | √ | √ | √ |
| BLH2-cluc | √ | √ | √ | √ | X | √ | √ | √ | X | √ | X | √ |
|  |  | Singal strong |  | Signal medium |  | Singal low | X | No signal |  |  |  |  |
